# Synthesis and Systematic Study on the Effect of Different PEG Units on Stability of PEGylated, Integrin-αvβ6-Specific A20FMDV2 Analogues in Rat Serum and Human Plasma

**DOI:** 10.3390/molecules27144331

**Published:** 2022-07-06

**Authors:** Kuo-yuan Hung, Renata Kowalczyk, Ami Desai, Margaret A. Brimble, John F. Marshall, Paul W. R. Harris

**Affiliations:** 1 The School of Chemical Sciences, University of Auckland, 23 Symonds St, Auckland 1010, New Zealand; hungkuoyuan@gmail.com (K.-y.H.); m.brimble@auckland.ac.nz (M.A.B.); 2 Centre for Tumour Biology, Barts Cancer Institute-Cancer Research UK Centre of Excellence, Queen Mary University of London, Charterhouse Square, London EC1M 6BQ, UK; ami_desai89@hotmail.com; 3 Maurice Wilkins Centre for Molecular Biodiscovery, University of Auckland, Private Bag 92019, Auckland 1010, New Zealand; 4 The School of Biological Sciences, University of Auckland, 3A Symonds St, Auckland 1010, New Zealand

**Keywords:** A20FMDV2, αvβ6, integrin, PEG, stability

## Abstract

A20FMDV2 is a 20-mer peptide that exhibits high selectivity and affinity for the tumour-related αvβ6 integrin that can compete with extracellular ligands for the crucial RGD binding site, playing a role as a promising αvβ6-specific inhibitor for anti-cancer therapies. Unfortunately, the clinical value of A20FMDV2 is limited by its poor half-life in blood caused by rapid renal excretion and its reported high susceptibility to serum proteases. The incorporation of poly (ethylene glycol) chains, coined PEGylation, is a well-established approach to improve the pharmacokinetic properties of drug molecules. Here, we report a systematic study on the incorporation of a varying number of ethylene glycol units (1–20) into the A20FMDV2 peptide to establish the effects of PEGylation size on the peptide stability in both rat serum and human plasma. In addition, the effect of acetyl and propionyl PEGylation handles on peptide stability is also described. Selected peptide analogues were assessed for integrin-αvβ6-targeted binding, showing good specificity and activity in vitro. Stability studies in rat serum established that all of the PEGylated peptides displayed good stability, and an A20FMDV2 peptide containing twenty ethylene glycol units (PEG_20_) was the most stable. Surprisingly, the stability testing in human plasma identified shorter PEGs (PEG_2_ and PEG_5_) as more resistant to degradation than longer PEGs, a trend which was also observed with affinity binding to integrin αvβ6.

## 1. Introduction

The integrin αvβ6 is a member of the integrin family that is expressed at low levels in healthy adult epithelia but is up-regulated during foetal development and tissue remodelling such as wound healing [1,2]. Increasing evidence has shown that integrin αvβ6 also promotes tumour growth and metastatic progression of many types of cancer including oral squamous-cell carcinoma, lung, pancreatic, colon, breast and ovarian [3,4,5,6,7,8]. Ligand binding to integrin αvβ6 is, in part, achieved by binding to the arginine–glycine–aspartate (RGD) motif [9] on specific extracellular ligands such as fibronectin [10], tenascin [11], and the latency-associated peptide (LAP) that forms part of latent-transforming growth factor beta [12,13]. Studies have revealed that synthetic peptides containing a DLXXL (X = any amino acid) subsequence exhibit enhanced affinity to integrin αvβ6 over RGD extracellular ligands and exert minimal effects on other integrins [14,15]. These results present a promising opportunity for the development of αvβ6-specific inhibitors as anti-cancer agents.

A20FMDV2 (H_2_N-^1^NAVPNLRGDLQVLAQKVAR^20^T-OH) is a 20-residue peptide derived from the viral protein of foot-and-mouth disease virus [16,17,18,19]. This peptide has been shown to exhibit high selectivity and affinity for integrin αvβ6 and can inhibit the binding of extracellular ligands to this integrin [20]. A20FMDV2 binds to integrin αvβ6 through the RGD motif of the ^7^RGDLQV^13^L fragment in which the two leucine residues assist the binding of the peptide to the receptor via hydrophobic interactions [21]. The binding of A20FMDV2 to integrin αvβ6 is further stabilised by an α-helix assembled from ^10^Leu-^17^Val and flanking *C*-terminal amino acid residues, thus contributing to the α-helix stability [16,18].

A20FMDV2 labelled with radioactive indium (^111^In) can be successfully used as an imaging agent for integrin αvβ6 in pulmonary fibrosis [12] and can also serve as a carrier for anti-cancer drugs to form a drug–peptide conjugate targeting integrin-αvβ6-expressing tumour cells [22]. Moreover, the safety of an A20FMDV2-based radioligand for PET studies for use in humans has been reported by Keat et al., when pre-clinical micro-dosing studies on safety, tolerability and immunogenicity were performed on four healthy human subjects using fluorine-18 (^18^F)-labelled 4-fluorobenzamine (FB)-A20FMDV2 [23]. This shows great promise for A20FMDV2-based ligands to be used in a clinical environment.

However, the use of unmodified A20FMDV2 as a therapeutic agent is limited by its very low half-life due to rapid renal excretion and high susceptibility to serum proteases [24]. We recently undertook a medicinal chemistry approach to improve the pharmacokinetics of A20FMDV2 by the substitution of individual amino acids and the modification of the *N-* and *C*-termini [25], while others have employed a decafluorobiphenyl-cyclisation strategy to afford cyclic A20FMDV2 variants that are more stable that their linear counterparts, whilst retaining αvβ6 integrin binding [26].

Another commonly employed modification to improve the pharmacokinetic properties of drug molecules is the incorporation of poly (ethylene glycol) (PEG) chains, a process known as PEGylation [27,28]. PEGs are non-toxic, water-soluble and FDA-approved biopolymers that can minimise the renal clearance and proteolytic degradation of conjugated molecules [29,30]. Synthetic PEGs of large sizes are usually supplied as polydisperse mixtures, affecting batch to batch repeatability during manufacture [31,32]. There is also some evidence that the conjugation of large-molecular-weight PEGs to proteins and biopharmaceuticals increases immunogenicity [33,34]. Nevertheless, PEGylation is a viable option to enhance the pharmaceutical and pharmacological profile of biologically active compounds, and many successful pharmaceuticals on the market are PEGylated [29,30,35,36].

**Figure 1:**
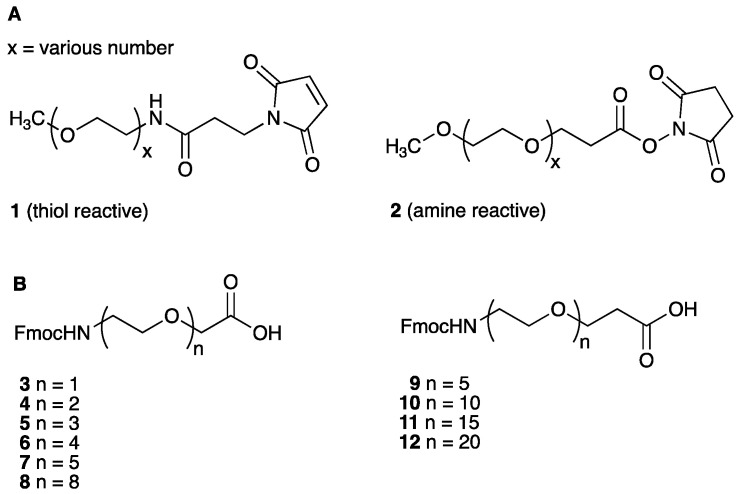
(**A**) Examples of common PEGylating reagents. (**B**) Structure of commercially available PEGs containing an acetyl handle (**3**–**8**) or a propionyl handle (**9**–**12**); n = number of ethylene glycol units.

Discrete PEG units such as **1** and **2** (Figure 1A) are readily available and are equipped with an appropriate functional group to permit site-selective PEGylation on amino acid functional groups such as thiols and amines. However, these reagents react indiscriminately with every reactive functional group present on a peptide or protein.

Well-defined PEGylating reagents **3**–**8** and **9**–**12**, respectively (Figure 1B, n = 1–20), are commercially obtainable, monomeric, and bifunctional, hence they can be condensed with the *N*-terminal amine or side-chain amine of a synthetic peptide. This allows the site-specific incorporation of PEGs via the formation of a stable amide bond and permits the multiple, controlled introduction of PEG units, thereby providing a homogenous PEGylated product.

The previous reports on the PEGylation of A20FMDV2 detail the incorporation of large PEG moieties, namely a PEG_28_ unit covalently attached to the peptide sequence to separate different chemical motifs such as radiotracers within A20FMDV2 [24,37,38,39,40].

Studies on A20FMDV2-based position emission tomography (PET) radiotracers incorporating 4-[^18^F]-fluorobenzoic acid ([^18^F]-FBA) and PEGs have resulted in beneficial effects of PEGylation on affinity, selectivity and enhanced stability [24,39]. It has been reported that the number and location of PEG_28_ moieties within the A20FMDV2 peptide chain (either *C*- or *N*- terminus) have different effects on binding to integrin αvβ6 and peptide metabolic stability [24,39].

One study established that selectivity and affinity to integrin αvβ6 for both [^18^F]-FBA-PEG_28_-A20FMDV2 and [^18^F]-FBA-(PEG_28_)_2_-A20FMDV2 is not compromised by the incorporation of PEG moieties to A20FMDV2 [24]. Importantly, it was revealed that pharmacokinetic characteristics, measured by the efficiency in tumour retention and washout from the pancreas, was more favourable for αvβ6-targeted pancreatic tumour detection in vivo in a BxPC-3 mouse model [24] using an analogue incorporating only one PEG_28_ unit. It was also noted that *N*-terminal A20FMDV2 peptide PEGylation improved the metabolic stability of the construct whilst *C*-terminal PEGylation increased tumour targeting [39]. These findings suggest that the size and number of the PEG unit itself as well as the location of it within the peptide sequence are important factors when designing PEGylated analogues of A20FMDV2.

In a subsequent study, in vitro and in vivo evaluation of 4,7,10-tetraazacyclododecane-1,4,7,10-tetraacetic acid (DOTA)- or 4,11-bis(carboxymethyl)-1,4,8,11-tetraazabicyclo [6.6.2]hexadecane (CB-TE2A)-modified A20FMDV2 analogues incorporating PEG_28_ showed high affinity and selectivity to αvβ6 integrin, proving a beneficial effect of PEG_28_ as a spacer between the Cu-chelating DOTA/CB-TEA2 and the peptide itself; importantly, significantly increased specific binding to cells expressing integrin αvβ6 was noted, compared to the analogue lacking PEG_28_ [37].

More recent studies by Huynth et al., employed PEG_28_-A20FMDV2-PEG_28_, which was *N*-terminally conjugated to either DOTA or 3,6,9,15-tetra-azabicyclo[9.3.1]pentadeca-1(15),11,13-triene-4-*S*-(4-isothiocyanatobenzyl)-3,6,9-triacetic acid (PCTA) for subsequent radiolabelling with [^64^Cu] to be used in the imaging or therapy of cancer [41]. The in vitro and in vivo evaluation of the probes’ affinity to αvβ6 in CaSki and BxPC-3 cells, tumour uptake in CaSki and BxPC-3 tumour-bearing mice, and stability in human serum was performed. Promising results for both of the tested probes were reported; however, further optimisation to improve tumour-to-normal-tissue ratios are needed [41].

In another study by the group, gallium-68 ([^68^Ga])-DOTA-PEG_28_-A20FMDV2-PEG_28_ and albumin binding, A20FMDV2-based peptides for the subsequent radiolabelling with lutetium-177 (^177^Lu), namely Evans Blue azo dye (EB)-PEG_28_-A20FMDV2-PEG_28_ and 4-(*p*-iodophenyl)butyric acid (IBA)-PEG_28_-A20FMDV2-PEG_28_, were tested for the αvβ6-specific cancer imaging and therapy [42]. It was shown that the enrichment of the probe with albumin binders increased the blood circulation time, but at the same time non-desired off-target radioactivity accumulation was also observed. The inhibition of tumour growth in mice was also observed; however, toxicity, especially for the Evans Blue azo-dye-enriched analogue, proved to be problematic. Further optimisation of the probes is anticipated to address safety and reduce non-desired normal tissue uptake [42].

Hausner et al., reported that for an A20FMDV2-based radiotracer incorporating a shorter PEG unit (PEG_7_), good stability in rat serum was observed. Unfortunately, compromised tumour uptake was noted in vivo, although this was attributed to the increased lipophilicity of the analogue that incorporated the [^18^F] prosthetic group via copper-free azide–alkyne [3+2]-dipolar cycloaddition [43,44,45] using the hydrophobic azadibenzocyclo-octyne (ADIBO) synthon ([^18^F]FBA-(CH_2_)_5_-ADIBO-N_3_-PEG_7_-A20FMDV2 construct), and not due to the smaller PEG unit [46].

Importantly, [^18^F]-FBA-PEG_28_-[16-Arg]A20FMDV2-PEG_28_ has been recently used in human imaging studies in patients diagnosed with breast, colon, lung or pancreatic cancer, proving to be a promising agent in the early detection of these malignancies due to the high affinity and selectivity for integrin αvβ6 [47]. Additionally, a significant improvement in overall pharmacokinetics was noted compared to the previously reported analogues, including those lacking PEG units [47].

Taken together, the above results demonstrated a therapeutic potential and an improved half-life of A20FMDV2 when either a PEG_28_ or PEG_7_ was incorporated. It should be noted that Li et al., have highlighted the risks associated with higher-molecular-weight PEGylation (5 kDa to 20 kDa) to potentially elicit an immunological response [33].

Many reports exist detailing the positive effect of PEGylation on A20FMDV2 stability when using either larger PEGs or significantly smaller ones (namely PEG_28_, bis-PEG_28_ and PEG_7_). To our best knowledge, no data have been reported thus far where the PEGylation impact on A20FMDV2 stability was investigated in a systematic manner or when using a variety of PEGs other than those that were already reported. The information gained when such studies are performed would be of high relevance to further expand understanding of the PEGylation pattern on A20FMDV2 stability, which could subsequently improve the therapeutic potential of the already clinically relevant A20FMDV2 peptide.

Prompted by the above results, we describe here a systematic study on the effect of increasing the PEGylation of A20FMDV2 as assessed by the measurement of the stability in both rat serum and human plasma, and changes in the specificity and affinity to integrin αvβ6.

Our aim was to identify the shortest PEG unit that improves the A20FMDV2 half-life without compromising its targeting ability to integrin αvβ6.

The effect of two different handles, via which PEG units were incorporated into the peptide, namely an acetyl or a propionyl handle, on A20FMDV2 stability was also studied.

Selected PEGylated A20FMDV2 analogues (PEG_2_–PEG_20_), were subsequently tagged with d-biotin; additionally, diethylenetriaminepenta-acetic acid (DTPA) was incorporated at the *N*-terminus to allow radiolabelling with indium-111 ([^111^In]). The stability in both rat and human plasma and the specificity and affinity to A375Pβ6 cells (expressing integrin αvβ6) of these compounds using flow cytometry was then assessed. We established that an A20FMDV2 analogue incorporating only five ethylene glycol units (PEG_5_) displayed preferential characteristics to the non-PEGylated peptide when considering the combined data on human plasma stability and binding ability to integrin αvβ6.

A schematic representation of all the analogues synthesised and tested during the studies is summarised in Table 1.

## 2. Results and Discussion

### 2.1. Synthesis and Stability Evaluation of A20FMDV2 (***13***) and PEGylated A20FMDV2 Analogues (***14***–***23***) in Rat Serum

PEGylation could, in theory, be carried out at either the *N*- or *C*- terminal region of A20FMDV2, or at the side-chain amino group of the native ^16^Lys residue. It has been reported that the modification of ^16^Lys may afford a peptide with reduced relative activity, most likely due to the disruption of the α-helical region [25]; additionally, *N*-terminal PEGylation using PEG_28_ was shown to be beneficial in improving A20FMDV2 stability over *C*-terminal PEGylation [39].

Thus, the focus was to establish the effect of the number of ethylene glycol units introduced at the *N*-terminus of A20FMDV2 peptide on its stability in rat serum. 

Commercially available 9-fluorenylmethoxycarbonyl (Fmoc)-protected building blocks **3**–**12** were used to amidate the *N*-terminus of A20FMDV2, generating analogues enriched in increasing numbers of ethylene glycol units (1, 2, 3, 4, 5, 8) via an acetyl spacer **14**–**19**. Analogues incorporating 5, 10, 15, and 20 ethylene glycol units via propionyl handle **20**–**23** were similarly prepared. The stability of the synthetic PEGylated analogues **14**–**23** was then probed in rat serum and compared to the native, non-PEGylated control A20FMDV2 (**13**).

**Scheme 1:**
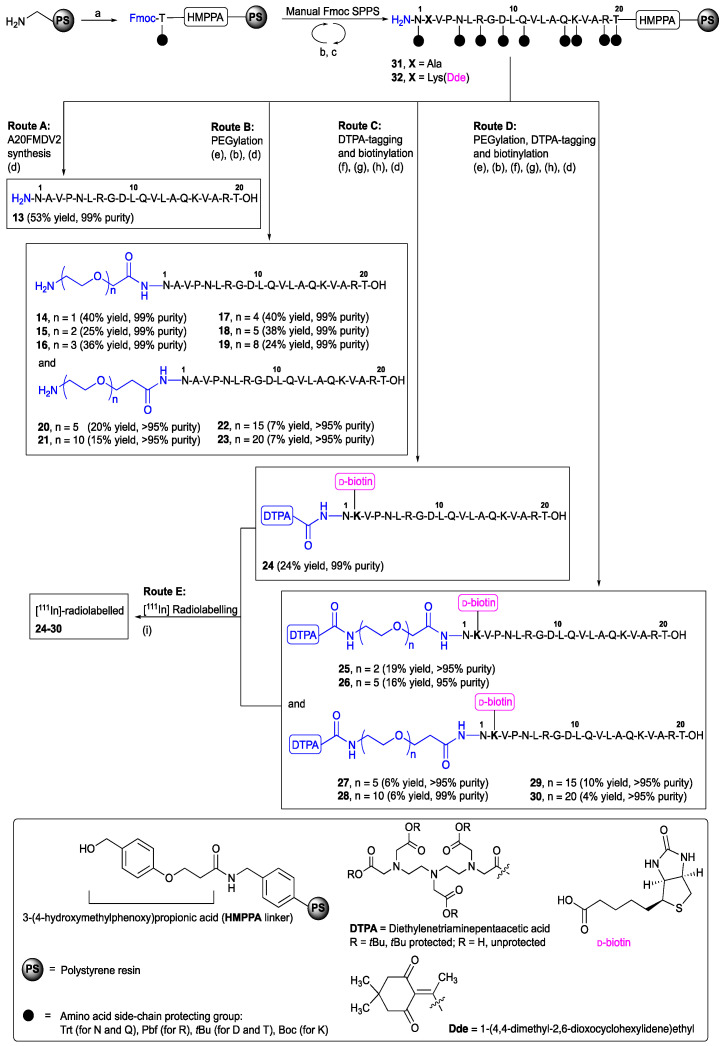
Synthesis of native A20FMDV2 (**13**), PEGylated analogues **14**–**23**, DTPA-tagged A20FMDV2 (**24**), and DTPA-tagged and PEGylated analogues **25**–**30**. Reagents and conditions: (a) Fmoc-Thr(*t*Bu)-HMPPA, DIC, DMF/CH_2_Cl_2_ (1:9, *v*:*v*), rt, 3 h; (b) 20% piperidine/DMF (*v*:*v*), rt, 1 × 5 min + 1 × 10 min; (c) Fmoc-AA-OH, HBTU, DIPEA, DMF, rt, 30 min; (d) TFA, *i*Pr_3_SiH, DODT, H_2_O, rt, 3 h; (e) respective PEGylating reagent (**3–12**), HBTU, DIPEA, DMF, rt, 30 min; (f) DTPA(O*t*Bu), HBTU, DIPEA, DMF, rt, 30 min; (g) 2% hydrazine hydrate/DMF (*v*:*v*), rt, 1 × 5 min + 1 × 10 min; (h) D-biotin, HBTU, DIPEA, NMP, rt, 30 min; (i) 3 MBq Indium [^111^In]Cl/nmol, 1 M ammonium acetate, pH 5.5, rt, 30 min.

The non-PEGylated control **13** and PEGylated A20FMDV2 peptides **14**–**23** were assembled at a 0.1 mmol scale using aminomethyl polystyrene resin (0.8 mmol/g) synthesised in house [48,49] and manual Fmoc/*tert*-butyl solid-phase peptide synthesis (Fmoc/*t*Bu SPPS) conditions as described previously and shown in Scheme 1 [25].

To synthesise PEGylated A20FMDV2 analogues **14**–**23**, the resin-bound peptide **31** was amidated using the suitable PEGylating reagent **3**–**12**, namely Fmoc-NH-(CH_2_-CH_2_-O)_n_-CH_2_-COOH (n = 1, 2, 3, 4, 5, 8) or Fmoc-NH-(CH_2_-CH_2_-O)_n_-CH_2_-CH_2_-COOH (n = 5, 10, 15, 20) under HBTU/DIPEA activation (30 min, rt) followed by the final Fmoc protecting group removal (20% piperidine) prior to the TFA-mediated release of the peptide from the resin Route B). Peptides **14**–**23** were recovered in >95% purity following purification by RP-HPLC (see Supplementary Materials).

The stability assay of the native A20FMDV2 peptide (**13**) and PEGylated derivatives (**14**–**23**) was then carried out in rat serum at 37 °C, following previously published procedures [46], and monitored by RP-HPLC over 48 h).

**Figure 2:**
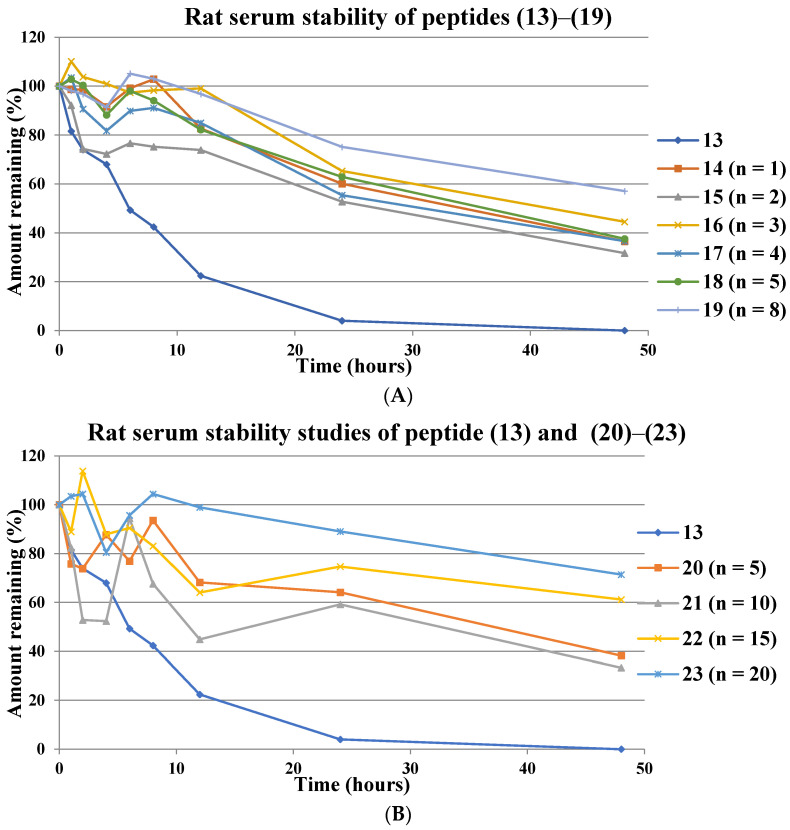
Detected levels (RP-HPLC) of A20FMDV2 (**13**) and PEGylated analogues (**14**–**19**), with ethylene glycol units attached via an acetyl handle (**A**) and propionyl handle (**B**), that remained over a period of 48 h in rat serum at 37 °C; n = number of ethylene glycol units in the PEG chain. Data expressed as percentage of peptide remaining over a period of 48 h.

The native peptide (**13**) was almost completely degraded after 24 h and was undetected at 48 h. For all the PEGylated peptides **14**–**19** where the PEGs were attached to A20FMDV2 via an acetyl spacer, proteolytic degradation was slower with >30% remaining intact after 48 h (Figure 2A). However, there was no clear trend in that increasing PEGylation did not necessarily result in an increase in peptide stability. Analogue **19** comprising eight ethylene glycol units (PEG_8_) proved to be the most stable in this series with more than 70% and 58% of the peptide intact at 24 h and 48 h, respectively.

In the PEGylated series **20**–**23** where a propionyl group was used to space PEGs from A20FMDV2, an improvement in stability compared to the native **13** was also observed (Figure 2B). Interestingly, except for the peptide analogue **21** comprising ten ethylene glycol units (PEG_10_), an increased number of ethylene glycol units (PEG_5_, PEG_15_, PEG_20_) improved the stability of the A20FMDV2 analogues **20**, **22**, and **23**. The A20FMDV2 analogue **23**, containing twenty ethylene glycol units (PEG_20_) was found to be the most stable across all the PEGylated analogues **14–23** overall, with close to 90% and >70% of the peptide remaining intact after 24 h and 48 h, respectively.

Analogues **18** and **20** comprising the same PEG_5_ component but different chemical handles, i.e., either acetyl (**18**) and propionyl (**20**), exhibited a similar degradation pattern in rat serum over the course of 24 h and 48 h (~60% for **18** and **20** after 24 h and ~40% for **18** and **20** after 48 h) suggesting that the length of the linker has no effect on degradation in rat serum.

### 2.2. Synthesis and Human Plasma Stability of [^111^In]-DTPA-[^2^Lys(d-biotin)]A20FMDV2 (***24***) and PEGylated [^111^In]-DTPA-[^2^Lys(d-biotin)]A20FMDV2 Analogues (***25***–***30***)

Previous studies on the stability of non-PEGylated A20FMDV2 in plasma suggested degradation in mouse serum [20] (50% remaining at 4 h), which significantly differs from the more relevant, clinically used environment of human plasma [25] (>75% remaining at 24 h). Thus, we established a degradation pattern for selected examples of PEGylated A20FMDV2 analogues in human plasma. In this instance, DTPA was incorporated at the *N*-terminus given the importance of A20FMDV2 as an imaging diagnostic using PET [50], or a single-photon emission computerized tomography (SPECT) that requires the metal chelating DTPA ligand. It was reported that the presence of DTPA on the A20FMDV2 peptide had no significant effect on the specificity and affinity of the peptide, but showed improved stability in mouse serum of [^111^In]-DTPA-[^2^Lys(d-biotin)]A20FMDV2 (50% intact peptide detected at 4 h) [20].

Additionally, to assess PEGylated A20FMDV2 peptide binding activity to the integrin αvβ6 using flow cytometry (see Section 2.3), d-biotin was incorporated via a side chain of the *N*^ε^-amino group of lysine-2, substituting the native alanine residue; this modification has previously been well tolerated [20,25,51].

Fmoc/*t*Bu SPPS of the non-PEGylated control **24** and PEGylated analogues of A20FMDV2 incorporating different numbers of ethylene glycol units (i.e., 2, 5, 10, 15, and 20), which were biotinylated at ^2^Lys and additionally tagged at the *N*-terminus with DTPA **25**–**30**, was therefore undertaken. In brief, for the preparation of the non-PEGylated analogue **24**, the resin-bound and side-chain-protected H_2_N-Asn(Trt)-Lys(Dde)-Val-Pro-Asn(Trt)-Leu-Arg(Pbf)-Gly-Asp(O*t*Bu)-Leu-Gln(Trt)-Val-Leu-Ala-Gln(Trt)-Lys(Boc)-Val-Ala-Arg(Pbf)-Thr(*t*Bu)-HMPPA-PS peptide **32** was directly reacted with *t*Bu-protected DTPA [52] using HBTU/DIPEA in DMF for 30 min at rt, Route C) [25]. For the synthesis of the PEGylated and DTPA-tagged variants **25**–**30**, an initial acylation of **32** with the corresponding Fmoc-NH-(CH_2_-CH_2_-O)_n_-CH_2_-COOH (n = 2, 5, analogues **25** and **26**, respectively) or Fmoc-NH-(CH_2_-CH_2_-O)_n_-CH_2_-CH_2_-COOH (n = 5, 10, 15, and 20, analogues **27**–**30**, respectively), followed by an Fmoc-protecting-group removal by employing the previously described synthetic protocol for the preparation of **13**–**23** [25], was required, prior to amidation with *t*Bu-protected DTPA, Route D). This was then followed by *N*^ε^-1-(4,4-dimethyl-2,6-dioxocyclohexylidene)ethyl (Dde)-protecting-group removal on ^2^Lys (2% hydrazine hydrate/DMF, *v*:*v*) and subsequent attachment of d-biotin to the liberated free *N*^ε^ amino group using HBTU/DIPEA in 1-methyl-2-pyrrolidone (NMP) for 30 min at rt [25]. Finally, the TFA-mediated peptide cleavage from the resin with simultaneous protecting-group removal afforded the desired constructs **24**–**30** in good purity (95% or more) following purification by RP-HPLC (see Supplementary Materials).

The DTPA-tagged peptides **24**–**30** were radiolabelled using 3 MBq [^111^In] chloride/nmol in 1 M ammonium acetate buffer (pH 5.5) (see Methods for details), affording [^111^In] radiolabelled analogues **24**–**30**, Route E). The radiolabelling efficiency and the purity of the radiolabelled compound were analysed by RP-HPLC.

**Figure 3:**
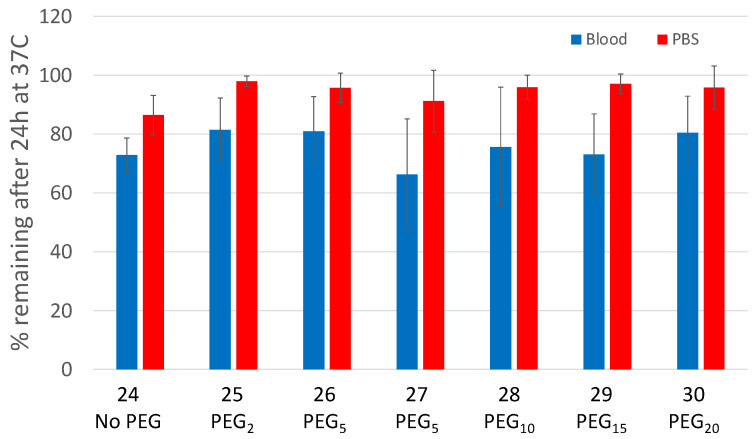
Stability of non-PEGylated and PEGylated [^111^In]-DTPA-[^2^Lys(d-biotin)]A20FMDV2 analogues (**24**–**30**) in human plasma. Data expressed as the amount of intact peptide relative to the input at time zero.

The [^111^In]-DTPA-A20FMDV2 control **24** and the corresponding PEGylated analogues [^111^In]-DTPA-NH-(CH_2_-CH_2_-O)_n_-CH_2_-[^2^Lys(d-biotin)]A20FMDV2 (n = 2, 5, **25** and **26**, respectively), and [^111^In]-DTPA-NH-(CH_2_-CH_2_-O)_n_-CH_2_-CH_2_-[^2^Lys(d-biotin)]A20FMDV2 (n = 5, 10, 15, and 20) **27**–**30,** respectively) were incubated in either human plasma or phosphate-buffered saline (PBS) at 37 °C for 24 h, and the samples were analysed using RP-HPLC, with the results reported as the percentage of peptide remaining after 24 h (Figure 3).

In accordance with our previous studies [25], the non-PEGylated peptide [^111^In]-labelled A20FMDV2 **24** remained with an average of 73% intact after 24 h in human plasma, which significantly differs from the previously reported stability of this peptide in mouse serum (50% of the peptide remained intact after 4 h) [20]. These data confirm our initial finding that A20FMDV2 is more stable in human plasma than mouse serum [25].

It has been observed in the past that the rates of enzymatic degradation of some peptides and their analogues differ depending on the biological media in which the stability testing was performed [53,54,55,56,57,58]. Among other factors, the observed differences in the rate of peptide breakdown are most likely due to the species-specific enzyme composition acting under different mechanisms and pathways [58]. Due to reactivity differences across media from human and animal species, it is paramount to use the biological medium of the greatest clinical importance for peptide evaluation. Similar to our observation for the A20FMDV2 peptide and the PEGylated analogues, Benuck and Marks [54] showed that rat serum proved to be more active than human serum in the stability studies of luteinizing hormone releasing hormone (LH-RH), thyrotropin releasing hormone (TRH) and somatostatin. Our finding further aligns with initial observations that A20FMDV2 is more stable in human plasma than mouse serum [20] and suggests human-derived media should be used for subsequent stability studies.

It was observed that the type of handle joining the PEG unit with A20FMDV2 had a strong effect on peptide stability when testing in human plasma. This was specifically noted for the [^111^In]-labelled A20FMDV2 analogues incorporating five ethylene glycol units (PEG_5_) **26** and **27**; after 24 h, the analogue **26** utilising an acetyl handle was 80% intact while analogue **27** bearing a propionyl group was 65% intact.

Importantly, it was noted that the A20FMDV2 analogues incorporating a smaller number of ethylene glycol units (i.e., PEG_2_ and PEG_5_) via acetyl handles (**25**, **26**) displayed a more beneficial effect on the stability in human plasma (around 80% of intact peptide detected after 24 h) over the analogues that included five or more ethylene glycol units (i.e., PEG_10_, PEG_15_, PEG_20_) introduced via a propionyl handle (**27**–**30**). Within the series of PEGylated analogues based on the propionyl handle (**27**–**30**), degradation showed a linear effect as the incorporation of an increased number of ethylene glycol units translated to a higher stability, as analogue **30** (PEG_20_) was ~80% intact after 24 h.

A comparison of the data obtained for the two different sera showed that the type of a handle (acetyl or propionyl) used to attach the PEG unit had no effect on peptide stability when testing was performed in rat serum (e.g., analogues **18**-acetyl and **20**-propionyl). The stability of the PEGylated analogues incorporating a larger number of ethylene glycol units (**20** to **23**, PEG_5_ to PEG_20_, all propionyl handles) in rat serum gradually increased with a PEG_20_ analogue being optimal, but in human plasma, the smaller PEG_2_ and PEG_5_ (with an acetyl handle) analogues performed the best.

### 2.3. Specificity and Affinity Evaluation for A375Ppuroβ6 and A375Ppuro Cells of DTPA-[^2^Lys(d-biotin)]A20FMDV2 (***24***) and DTPA PEGylated-[^2^Lys(d-biotin)]A20FMDV2 Analogues (***25***–***30***) Using Flow Cytometry

To assess specificity and affinity of the PEGylated A20FMDV2 analogues **25**–**30** for integrin αvβ6, we used A375Pβ6 cells, which only express integrin αvβ6, and compared this to the binding to A375Ppuro, which contains equal amounts of four RGD-binding integrins (i.e., α5β1, αvβ3, αvβ5, αvβ8) [59].

**Figure 4:**
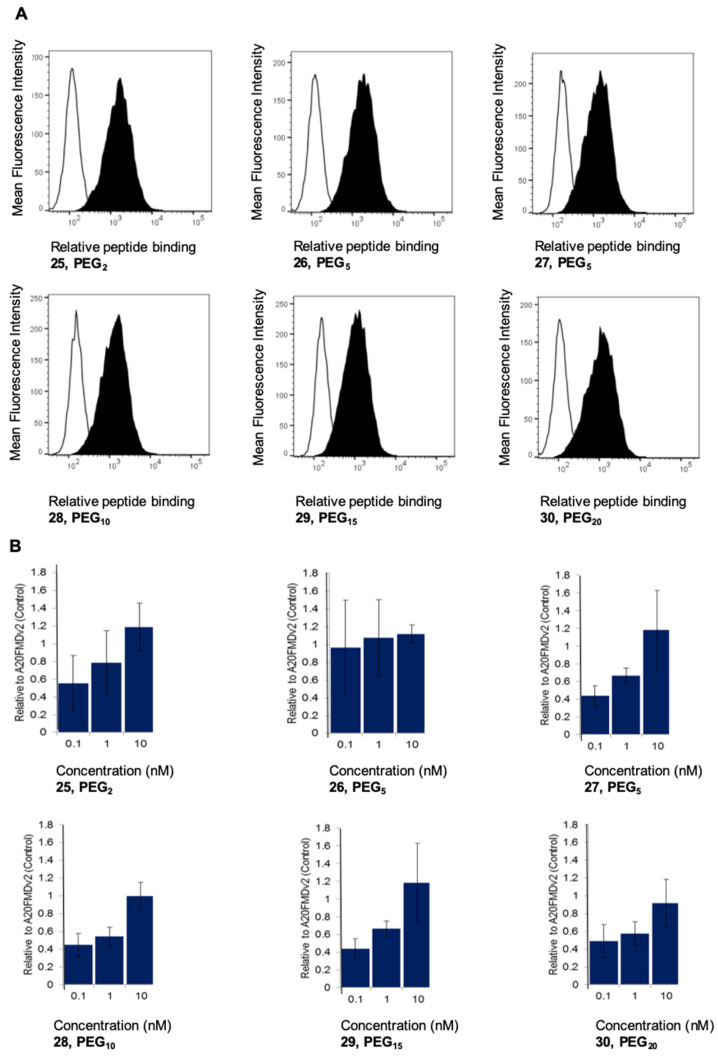
(**A**) Specificity of PEGylated DTPA-[^2^Ala to Lys(d-biotin)]A20FMDV2 analogues (**25**–**30**) for integrin αvβ6 by flow cytometry; the binding of the peptide (1000 nM) to αvβ6-negative A375Ppuro (white histogram) versus αvβ6-positive A375Pβ6 cells (black histogram). (**B**) Relative activity of PEGylated DTPA-[^2^Ala to Lys(d-biotin)]A20FMDV2 analogues (**25**–**30**) for αvβ6 measured by the cell-bound fraction at various concentrations. Data show the binding relative to the same concentration of non-PEGylated DTPA-NK(d-biotin)VPNLRGDLQVLAQKVART as assessed by flow cytometry. Data show mean of three experiments +/− SD.

The PEGylated A20FMDV2 analogues **25**–**30** were added at a 1000 nM concentration to A375Ppuro or A375Pβ6 cells and binding was measured using flow cytometry. It was shown that none of the peptides bound to the αvβ6-negative A375Ppuro cells, but all the peptides bound equally well to the αvβ6-expressing A375Pβ6 cells, thus confirming that the PEGylated variants retained their absolute specificity for integrin αvβ6 (Figure 4A).

To assess the affinity of PEGylated A20FMDV2 for integrin αvβ6, concentrations of 0.1 nM, 1 nM and 10 nM were compared to the binding of non-PEGylated DTPA-[^2^Lys(d-biotin)]A20FMDV2 (**24**).

Across all the concentrations, no loss of activity equated to a value of 1. At the 0.1 nM and 1 nM concentrations, most of the PEGylated peptides lost some affinity compared with the parent peptide (i.e., had a relative value less than 1), particularly those with fifteen and twenty ethylene glycol units (PEG_15_ and PEG_20_, **29** and **30**, respectively; Figure 4B). However, at the 10 nM concentration, all the PEGylated analogues **25**–**30** achieved statistically (Anova) similar activities to the parent peptide, revealing that all the PEGylated peptides had good affinity. These data demonstrate that all the PEGylated A20FMDV2 peptides retained complete specificity for αvβ6, but there was some variation in activity at very low concentrations of the peptide.

Hausner et al. [39] reported changes in the in vitro activity of A20FMDV2 that were dependent on the positioning and number of PEG_28_ units within the A20FMDV2 peptide sequence. The addition of one PEG_28_ to the *N*-terminus, or to both the *N*- and *C*-termini, retained more activity in vitro than when adding two PEG_28_ units in series to the *N*-terminus. It is known that A20FMDV2 binds to integrin αvβ6 through both the RGD motif of the ^7^RGDLQV^13^L sequence and the two leucine residues [16,17,18]. This binding of A20FMDV2 to αvβ6 is stabilised by an α-helix that is assembled from a *C*-terminal peptide motif presenting the two non-adjacent leucines as a hydrophobic binding interface [16,17,18]. The changes in activity observed by Hausner et al. [39] and ourselves (Figure 4) suggest that determining the positioning and number of PEG units within the parent (A20FMDV2) scaffold peptide is critical to minimise the disruption of the ability of the peptide to assume its active conformer. In the work reported here, all the PEGylated A20FMDV2 peptides lost some activity when compared to the native A20FMDV2, but the longer PEGs PEG_15_ and PEG_20_ were less tolerated.

## 3. Conclusions

A20FMDV2 is a promising lead peptide for cancer treatment as it exhibits high specificity and affinity for the tumour-related integrin αvβ6 and can inhibit the activation of this integrin by competing with extracellular ligands for the key binding site in integrin αvβ6. However, the short half-life of A20FMDV2 in blood, caused by the rapid renal excretion and serum proteolytic degradation, suggests that structural modification is required to overcome these limitations. The clinical potential of the PEGylated A20FMDV2 peptide incorporating larger PEG moieties (bis-PEG_28_) has already been realized [47]. It has been reported in the past that the presence of either bis-PEG_28_, PEG_28_ or PEG_7_ within the A20FMDV2 peptide sequence has a positive effect on the peptide pharmacokinetics [24,37,38,39,40,41,42,46]. The current studies outlined here fulfil the gap in the knowledge where effects of other PEG moieties (PEG_1_-PEG_20_) on A20FMDV2 stability have been investigated with the aim to identify the shortest ethylene glycol repeat that improves peptide stability. The information gained is of great importance to further advance future directions within the A20FMDV2-based drug-discovery pipeline.

Herein, a systematic study was performed on the effect of a discrete number of ethylene glycol units (PEG_1_- PEG_20_) incorporated in an A20FMDV2 peptide at the *N*-terminus, as well as the choice of the linker (acetyl and propionyl) to couple the corresponding PEG units to the A20FMDV2 peptide, on the peptide stability in rat serum (**14**–**23**) and human plasma (**25**–**30**). All the peptides could be successfully accessed using Fmoc/*t*Bu SPPS. All the PEGylated (PEG_1_-PEG_20_) A20FMDV2 peptide analogues were less susceptible to proteolytic degradation than the native A20FMDV2 peptide in rat serum.

In human plasma, the stability of the PEGylated A20FMDV2 peptide analogues differed significantly from rat serum. The A20FMDV2 analogues comprising a smaller number of ethylene glycol units (PEG_2_ and PEG_5_) were highly stable in human plasma, while the PEGylated PEG_20_ A20FMDV2 peptide proved the most stable in rat serum. We concluded that stability differences in both media underline the importance of the choice of media to more precisely mimic a natural environment of the candidates with clinical potential. It is now evident that human-derived plasma should be the media of choice.

The effect of the chemical handle joining the PEGs with the A20FMDV2 peptide was more pronounced when the degradation studies were performed in human plasma. A PEG_5_-linked acetyl A20FMDV2 was superior to a PEG_5_ propionyl linkage in human plasma, which was more than 80% intact after 24 h; the corresponding propionyl linked PEG_5_ was approximately 65% intact after the same time period. This effect on peptide stability was not observed when the degradation studies were performed in rat serum.

The above observation was also reflected in the binding of the PEG_5_ylated A20FMDV2 peptide to the integrin αvβ6, as the use of the acetyl handle (**26**) showed improved binding at 0.1 nM and 1 nM over its propionyl counterpart (**27**). Taking all the above data into consideration, the PEG_5_-A20FMDV2 analogue **26** containing an acetyl handle had excellent stability in human plasma and retained selectivity and nanomolar affinity for integrin αvβ6.

Subsequent studies will be undertaken in the future to evaluate promising analogues on cell binding and internalization as well as tumour and normal tissue uptake, and to compare their therapeutic potential with currently existing bis-PEG_28_ A20FMDV2 analogue. These studies will further assess the prospect of simplified PEG modifications on the clinical potential of A20FMDV2 using PEG units that are shorter than those currently employed.

## 4. Materials and Methods

### 4.1. Chemistry

All reagents were purchased as reagent grade and used without further purification. The 9-Fluorenylmethoxycarbonyl (Fmoc)-protected amino acids were purchased from CS Bio (Shanghai, China). Fmoc-amino acids were supplied with the following side-chain protection: Fmoc-Asn(Trt)-OH (Trt = triphenylmethyl), Fmoc-Arg(Pbf)-OH (Pbf = 2,2,4,6,7-pentamethyldihydrobenzofuran-5-sulfonyl), Fmoc-Asp(O*t*Bu)-OH (*t*Bu = *tert*-butyl), Fmoc-Gln(Trt)-OH, Fmoc-Lys(Boc)-OH (Boc = *tert*-butyloxycarbonyl), Fmoc-Thr(*t*Bu)-OH. Fmoc-Thr(*t*Bu)-3-(4-hydroxymethylphenoxy) propionic acid (HMPPA) was purchased from PolyPeptide (Strasbourg, France). Fmoc-NH-(CH_2_-CH_2_-O)_n_-CH_2_-COOH (n = 1–5, and 8) and Fmoc-NH-(CH_2_-CH_2_-O)_n_-CH_2_-CH_2_-COOH (n = 5, 10, 15, and 20) were purchased either from ChemPep Inc. (Wellington, FL, USA) or Peptides International Inc. (Louisville, KY, USA). Fmoc-Lys(Dde)-OH (Dde = 1-(4,4-dimethyl-2,6-dioxocyclohexylidene)ethyl), *O*-(benzotriazol-1-yl)-*N*,*N*,*N*′,*N*′-tetramethyluronium hexafluorophosphate (HBTU) and *N*,*N*′-diisopropylcarbodiimide (DIC) were sourced from GL Biochem (Shanghai, China). *N*,*N-*Diisopropylehylamine (DIPEA), piperidine, 2% hydrazine hydrate, d-biotin, triisopropylsilane (*i*Pr_3_SiH), 2,2′-(ethylenedioxy)diethanethiol (DODT), formic acid, diethyl ether (Et_2_O), caffeine, 1-methyl-2-pyrrolidone (NMP), ammonium acetate, ethylenediaminetetraacetic acid (EDTA), phosphate-buffered saline (PBS), ninhydrin, phenol, and potassium cyanide (KCN) were purchased from Sigma-Aldrich (St. Louis, MO, USA). Trifluoroacetic acid (TFA) was purchased from Halocarbon (River Edge, NJ, USA). Dichloromethane (CH_2_Cl_2_) and *N*,*N*-dimethylformamide (DMF), were purchased from ECP Ltd. (Auckland, New Zealand). Acetonitrile (MeCN) was purchased from Thermo Fisher Scientific (Waltham, MA, USA). Previously published procedures were used for the synthesis of aminomethyl polystyrene resin (0.8 mmol/g) [48,49] and diethylenetriaminepentaacetic acid *tetra tert*-butyl ester (DTPA(O*t*Bu)) [52]. Indium [^111^In] chloride was purchased from Tyco Healthcare UK Commercial Ltd. (Hampshire, UK).

### 4.2. General Procedure for Peptide Synthesis, Purification, and Analysis

Peptides **13–30** were assembled using manual Fmoc/tBu SPPS (0.1 mmol) using previously published procedures [25] and starting from aminomethyl polystyrene resin (0.8 mmol/g) synthesised in house. PEGylation was carried out using the following procedure: a solution of Fmoc-NH-(CH_2_-CH_2_-O)n-CH_2_-COOH (n = 1–5, and 8, 3–8) or Fmoc-NH-(CH_2_-CH_2_-O)_n_-CH_2_-CH_2_-COOH (n = 5, 10, 15, and 20, 9–12) (5 equiv.), HBTU (4.75 equiv.) and DIPEA (10 equiv.) in DMF (1 mL) was added to the resin. After agitating the mixture at rt for 30 min, the solution was drained, and the resin washed with DMF (3 × 5 mL).

After the last step of synthesis, the peptidyl resin was washed thoroughly with CH_2_Cl_2_ and dried under the flow of nitrogen. Resin cleavage with simultaneous removal of the amino acid side-chain-protecting groups was undertaken by incubating the resin in TFA/*i*Pr_3_SiH/H2O/DODT (*v*/*v*/*v*/*v*; 94/1/2.5/2.5) cleavage cocktail (5 mL) for 3 h at room temperature (rt). The crude peptides were precipitated and triturated with cold diethyl ether, isolated (centrifugation), dissolved in 50% MeCN (aq) containing 0.1% TFA and lyophilized, prior to their final purification by reverse-phase high-performance liquid chromatography (RP-HPLC).

Peptide purification was performed using a Waters 600 System with a Waters 2487 dual wavelength absorbance detector using a Waters (Milford, MA, USA) Xterra^®^ Prep MS C18 10 μm; 19 × 300 mm preparative column at a flow rate of 10 mL/min. Gradient systems were adjusted according to the elution and peak profiles obtained from the analytical RP-HPLC chromatograms. Fractions were collected, analysed by either RP-HPLC or ESI-MS, pooled and lyophilised. Isolated peptide yields were calculated based on 0.1 mmol synthesis. Analytical RP-HPLC was performed using Thermo Scientific (Waltham, MA, USA) Dionex Ultimate U3000 system (flow rate of 1 mL/min), and a Waters (Milford, ME, USA) Xterra^®^ C18 5 μm; 4.6 × 150 mm analytical column, using a linear gradient of 5%B to 95%B over 30 min, ca. 3%B per minute. The solvent system used was A (0.1% TFA in H_2_O) and B (0.1% TFA in MeCN) with detection at 210 nm. Direct infusion ESI-MS were recorded on an Agilent Technologies (Santa Clara, CA, USA) 1120 Compact LC connected to an in-line Hewlett Packard (Palo Alto, CA, USA) 1100MSD spectrometer. Samples were introduced using direct flow injection at 0.2 mL/min into an ESI source in the positive mode. The solvent system used was 0.1% formic acid in H_2_O and 0.1% formic acid in MeCN (1:1, *v*:*v*). Major and significant fragments were quoted in the form x m/z (mass to charge ratio).

Peptide radiolabelling was performed according to previously published procedures [20].

## 5. Biological Studies

### 5.1. Cell Lines

A375Ppuro (αvβ6-negative) and A375Pβ6 (αvβ6-positive) cell lines were grown as monolayers in Dulbecco’s Modification of Eagles Medium (DMEM; Gibco, Scotland) supplemented with 10% *v*/*v* foetal calf serum (Sigma) at 37 °C in a 100% humidified incubator, 8% *v*/*v* CO_2_. All chemicals were purchased from Sigma Aldrich unless otherwise specified.

### 5.2. Preparation of Peptide

All peptides were dissolved in 0.1%TFA/water to prepare 1 mM stock solutions, which were aliquoted and stored at −20 °C. Thawed samples were diluted directly into cell growth medium.

### 5.3. Biological Assessment of PEGylated Peptides

To assess specificity and relative activity of peptides, we incubated 2 × 10^5^ A375Ppuro or A375Pβ6 cells on wet ice with 1000, 100, 10, 1, 0.1 and 0 nM of each peptide prepared in DMEM supplemented with 0.1% NaN_3_ and 0.1% bovine serum albumin (0.1/0.1). After 30 min, the unbound peptides were washed away with 0.1/0.1 by centrifugation (twice for 3 min 120× *g*). Mouse anti-biotin antibody (Jackson ImmunoResearch Laboratories Inc., West Grove, PA, USA) was added at 1:100 on ice and again washed after 30 min. Rabbit-anti mouse IgG conjugated to Alexafluor 488 (1:250 final dilution; Molecular Probes, Eugene, OR, USA) was added for an additional 30 min on ice before a final washing and resuspension in 500 μL of 0.1/0.1. Samples were analysed by flow cytometry (FACScan, Becton-Dickinson, Franklin Lakes, NJ, USA). Geometric Mean Fluorescence Intensity (MFI) of 10,000 cells was collected. To assess relative changes in affinity, the above experiment was repeated with at least 3 biological repeats and the results were expressed by comparison with Peptide **24** (DTPA-NK(biotin)VPNLRGDLQVLAQKVART), which was run at the same time.

## 6. Rat Serum Stability

The stability assay of A20FMDV2 (**13**) and its PEGylated derivatives (**14**)–(**23**) was performed according to a published protocol [46]. Each peptide was dissolved in a 0.1 M sodium phosphate buffer solution (2.7 mL) (pH 7.4) containing 25% rat serum to yield a final peptide concentration of 20 μM. The resultant solution was mixed, and nine aliquots (270 μL each) were taken and distributed into 1.5 mL Eppendorf vials. The tubes were shaken at 37 °C for different time periods (t = 0 h, 1 h, 2 h, 4 h, 6 h, 8 h, 12 h, 24 h and 48 h), and a 3-fold excess of cold MeCN (810 μL) was added to the vials after each time point and the mixture was placed at 4 °C for 1 h. Caffeine (90 μL of a 0.0002 mg/mL solution in 0.1% TFA in H_2_O), which was used as an internal standard, was added to the mixture, and the mixture was centrifuged at 14,500 rpm for 30 min. The supernatant was transferred to a clean 1.5 mL Eppendorf vial, and the solvent was reduced to half of the original volume using a Speed-Vac concentrator and then lyophilised. The sample was re-dissolved in 0.1% TFA in H_2_O solution (150 μL), and a portion (80 μL) was analysed by RP-HPLC. The amount of peptide remaining at each time interval was determined as the percentage of the peptide signal, which is calculated as the ratio of the peptide’s peak area under the curve (AUC) to the caffeine AUC, relative to the signal at t = 0 h.

### Human Plasma Stability Assays

Human blood was taken from healthy volunteers in sodium heparin tubes and centrifuged at 2000× *g* for 10 min. Plasma (300 μL) was incubated with 7.5 MBq of radiolabelled peptide at 37 °C and 150 μL aliquots were analysed either immediately (t = 0) or after 24 h as follows: samples were treated with an equal volume of ice-cold MeCN, then mixed and centrifuged at 14,000 rpm for 5 min. The supernatants were collected and dried using a centrifugal evaporator for 10 min to remove MeCN. The residuum was filtered through a 0.22 μm filter and analysed by RP-HPLC. Radiopeptides were also incubated at 37 °C with PBS and analysed without further sample preparation.

## Figures and Tables

**Table 1:** Synthesized peptides during the studies: A20FMDV2 (**13**), DTPA-[^2^Ala to Lys(d-biotin]A20FMDV2 (**24**) and PEGylated peptide analogues [**N-term**]-**X1**-N-**X2**-VPNLRGDLQVLAQLVART-OH (**14**–**23**, **25**–**30**).

Compound	N-term.	X1	X2
**13** (A20FMDV2)	NH_2_	-	Ala
**14**	NH_2_	-CH_2_-CH_2_-O-CH_2_-CO-	Ala
**15**	NH_2_	-(CH_2_-CH_2_-O)_2_-CH_2_-CO-	Ala
**16**	NH_2_	-(CH_2_-CH_2_-O)_3_-CH_2_-CO-	Ala
**17**	NH_2_	-(CH_2_-CH_2_-O)_4_-CH_2_-CO-	Ala
**18**	NH_2_	-(CH_2_-CH_2_-O)_5_-CH_2_-CO-	Ala
**19**	NH_2_	-(CH_2_-CH_2_-O)_8_-CH_2_-CO-	Ala
**20**	NH_2_	-(CH_2_-CH_2_-O)_5_-CH_2_-CH_2_-CO-	Ala
**21**	NH_2_	-(CH_2_-CH_2_-O)_10_-CH_2_-CH_2_-CO-	Ala
**22**	NH_2_	-(CH_2_-CH_2_-O)_15_-CH_2_-CH_2_-CO-	Ala
**23**	NH_2_	-(CH_2_-CH_2_-O)_20_-CH_2_-CH_2_-CO-	Ala
**24**	DTPA-NH	-	Lys(d-biotin)
**25**	DTPA-NH	-(CH_2_-CH_2_-O)_2_-CH_2_-CO-	Lys(d-biotin)
**26**	DTPA-NH	-(CH_2_-CH_2_-O)_5_-CH_2_-CO-	Lys(d-biotin)
**27**	DTPA-NH	-(CH_2_-CH_2_-O)_5_-CH_2_-CH_2_-CO-	Lys(d-biotin)
**28**	DTPA-NH	-(CH_2_-CH_2_-O)_10_-CH_2_-CH_2_-CO-	Lys(d-biotin)
**29**	DTPA-NH	-(CH_2_-CH_2_-O)_15_-CH_2_-CH_2_-CO-	Lys(d-biotin)
**30**	DTPA-NH	-(CH_2_-CH_2_-O)_20_-CH_2_-CH_2_-CO-	Lys(d-biotin)

## Data Availability

Not applicable.

## Supplementary Materials

The following supporting information can be downloaded at: https://www.mdpi.com/article/10.3390/molecules27144331/s1: All characteristic data (RP-HPLC and MS) of the synthesized peptides **13–30.**

## References

[1] Breuss J.M., Gillett N., Lu L., Sheppard D., Pytela R. (1993). Restricted Distribution of Integrin Beta 6 MRNA in Primate Epithelial Tissues. J. Histochem. Cytochem..

[2] Breuss J.M., Gallo J., DeLisser H.M., Klimanskaya I.V., Folkesson H.G., Pittet J.F., Nishimura S.L., Aldape K., Landers D.V., Carpenter W. (1995). Expression of the Beta 6 Integrin Subunit in Development, Neoplasia and Tissue Repair Suggests a Role in Epithelial Remodeling. J. Cell Sci..

[3] Bates R.C., Bellovin D.I., Brown C., Maynard E., Wu B., Kawakatsu H., Sheppard D., Oettgen P., Mercurio A.M. (2005). Transcriptional Activation of Integrin Β6 during the Epithelial-Mesenchymal Transition Defines a Novel Prognostic Indicator of Aggressive Colon Carcinoma. J. Clin. Investig..

[4] Thomas G.J., Nyström M.L., Marshall J.F. (2006). αvβ6 Integrin in Wound Healing and Cancer of the Oral Cavity. J. Oral Pathol. Med..

[5] Elayadi A.N., Samli K.N., Prudkin L., Liu Y.-H., Bian A., Xie X.-J., Wistuba I.I., Roth J.A., McGuire M.J., Brown K.C. (2007). A Peptide Selected by Biopanning Identifies the Integrin αvβ6 as a Prognostic Biomarker for Nonsmall Cell Lung Cancer. Cancer Res..

[6] Hazelbag S., Kenter G.G., Gorter A., Dreef E.J., Koopman L.A., Violette S.M., Weinreb P.H., Fleuren G.J. (2007). Overexpression of the Alpha v Beta 6 Integrin in Cervical Squamous Cell Carcinoma Is a Prognostic Factor for Decreased Survival. J. Pathol..

[7] Bandyopadhyay A., Raghavan S. (2009). Defining the Role of Integrin Alphavbeta6 in Cancer. Curr. Drug Targets.

[8] Moore K.M., Thomas G.J., Duffy S.W., Warwick J., Gabe R., Chou P., Ellis I.O., Green A.R., Haider S., Brouilette K. (2014). Therapeutic Targeting of Integrin αvβ6 in Breast Cancer. J. Natl. Cancer Inst..

[9] Ruoslahti E. (1996). RGD and Other Recognition Sequences for Integrins. Annu. Rev. Cell Dev. Biol..

[10] Busk M., Pytela R., Sheppard D. (1992). Characterization of the Integrin Alpha v Beta 6 as a Fibronectin-Binding Protein. J. Biol. Chem..

[11] Prieto A.L., Edelman G.M., Crossin K.L. (1993). Multiple Integrins Mediate Cell Attachment to Cytotactin/Tenascin. Proc. Natl. Acad. Sci. USA.

[12] Munger J.S., Huang X., Kawakatsu H., Griffiths M.J.D., Dalton S.L., Wu J., Pittet J.-F., Kaminski N., Garat C., Matthay M.A. (1999). A Mechanism for Regulating Pulmonary Inflammation and Fibrosis: The Integrin αvβ6 Binds and Activates Latent TGF Β1. Cell.

[13] Annes J.P., Rifkin D.B., Munger J.S. (2002). The Integrin αvβ6 Binds and Activates Latent TGFβ3. FEBS Lett..

[14] Mateu M.G., Valero M.L., Andreu D., Domingo E. (1996). Systematic Replacement of Amino Acid Residues within an Arg-Gly-Asp-Containing Loop of Foot-and-Mouth Disease Virus and Effect on Cell Recognition. J. Biol. Chem..

[15] Kraft S., Diefenbach B., Mehta R., Jonczyk A., Luckenbach G.A., Goodman S.L. (1999). Definition of an Unexpected Ligand Recognition Motif for αvβ6 Integrin. J. Biol. Chem..

[16] Logan D., Abu-Ghazaleh R., Blakemore W., Curry S., Jackson T., King A., Lea S., Lewis R., Newman J., Parry N. (1993). Structure of a Major Immunogenic Site on Foot-and-Mouth Disease Virus. Nature.

[17] Jackson T., Sheppard D., Denyer M., Blakemore W., King A.M.Q. (2000). The Epithelial Integrin αvβ6 Is a Receptor for Foot-and-Mouth Disease Virus. J. Virol..

[18] DiCara D., Rapisarda C., Sutcliffe J.L., Violette S.M., Weinreb P.H., Hart I.R., Howard M.J., Marshall J.F. (2007). Structure-Function Analysis of Arg-Gly-Asp Helix Motifs in αvβ6 Integrin Ligands. J. Biol. Chem..

[19] Meecham A., Marshall J. (2021). Harnessing the Power of Foot-and-Mouth-Disease Virus for Targeting Integrin Alpha-v Beta-6 for the Therapy of Cancer. Expert Opin. Drug Discov..

[20] Saha A., Ellison D., Thomas G.J., Vallath S., Mather S.J., Hart I.R., Marshall J.F. (2010). High-resolution in vivo Imaging of Breast Cancer by Targeting the Pro-invasive Integrin αvβ6. J. Pathol..

[21] Dicara D., Burman A., Clark S., Berryman S., Howard M.J., Hart I.R., Marshall J.F., Jackson T. (2008). Foot-and-Mouth Disease Virus Forms a Highly Stable, EDTA-Resistant Complex with Its Principal Receptor, Integrin Alphvbeta6: Implications for Infectiousness. J. Virol..

[22] Firer M.A., Gellerman G. (2012). Targeted Drug Delivery for Cancer Therapy: The Other Side of Antibodies. J. Hematol. Oncol..

[23] Keat N., Kenny J., Chen K., Onega M., Garman N., Slack R.J., Parker C.A., Lumbers R.T., Hallett W., Saleem A. (2018). A Microdose PET Study of the Safety, Immunogenicity, Biodistribution, and Radiation Dosimetry of 18F-FB-A20FMDV2 for Imaging the Integrin αvβ6. J. Nucl. Med. Technol..

[24] Hausner S.H., Abbey C.K., Bold R.J., Gagnon M.K., Marik J., Marshall J.F., Stanecki C.E., Sutcliffe J.L. (2009). Targeted in vivo Imaging of Integrin αvβ6 with an Improved Radiotracer and Its Relevance in a Pancreatic Tumor Model. Cancer Res..

[25] Hung K.Y., Harris P.W.R., Desai A., Marshall J.F., Brimble M.A. (2017). Structure-Activity Relationship Study of the Tumour-Targeting Peptide A20FMDV2 via Modification of Lys16, Leu13, and N- and/or C-Terminal Functionality. Eur. J. Med. Chem..

[26] Cardle I.I., Jensen M.C., Pun S.H., Sellers D.L. (2021). Optimized Serum Stability and Specificity of an αvβ6 Integrin-Binding Peptide for Tumor Targeting. J. Biol. Chem..

[27] Abuchowski A., van Es T., Palczuk N.C., Davis F.F. (1977). Alteration of Immunological Properties of Bovine Serum Albumin by Covalent Attachment of Polyethylene Glycol. J. Biol. Chem..

[28] Abuchowski A., McCoy J.R., Palczuk N.C., van Es T., Davis F.F. (1977). Effect of Covalent Attachment of Polyethylene Glycol on Immunogenicity and Circulating Life of Bovine Liver Catalase. J. Biol. Chem..

[29] Alconcel S.N.S., Baas A.S., Maynard H.D. (2011). FDA-Approved Poly(Ethylene Glycol)–Protein Conjugate Drugs. Polym. Chem..

[30] Gupta V., Bhavanasi S., Quadir M., Singh K., Ghosh G., Vasamreddy K., Ghosh A., Siahaan T.J., Banerjee S., Banerjee S.K. (2019). Protein PEGylation for Cancer Therapy: Bench to Bedside. J. Cell Commun. Signal..

[31] van Witteloostuijn S.B., Pedersen S.L., Jensen K.J. (2016). Half-Life Extension of Biopharmaceuticals Using Chemical Methods: Alternatives to PEGylation. ChemMedChem.

[32] Turecek P.L., Bossard M.J., Schoetens F., Ivens I.A. (2016). PEGylation of Biopharmaceuticals: A Review of Chemistry and Nonclinical Safety Information of Approved Drugs. J. Pharm. Sci..

[33] Li B., Yuan Z., Hung H.-C., Ma J., Jain P., Tsao C., Xie J., Zhang P., Lin X., Wu K. (2018). Revealing the Immunogenic Risk of Polymers. Angew. Chem. Int. Ed..

[34] Shiraishi K., Yokoyama M. (2019). Toxicity and Immunogenicity Concerns Related to PEGylated-Micelle Carrier Systems: A Review. Sci. Technol. Adv. Mater..

[35] Harris J.M., Chess R.B. (2003). Effect of PEGylation on Pharmaceuticals. Nat. Rev. Drug Discov..

[36] Swierczewska M., Lee K.C., Lee S. (2015). What Is the Future of PEGylated Therapies?. Expert Opin. Emerg. Drugs.

[37] Hausner S.H., Kukis D.L., Gagnon M.K.J., Stanecki C.E., Ferdani R., Marshall J.F., Anderson C.J., Sutcliffe J.L. (2009). Evaluation of [64Cu]Cu-DOTA and [64Cu]Cu-CB-TE2A Chelates for Targeted Positron Emission Tomography with an αvβ6-Specific Peptide. Mol. Imaging.

[38] Hu L.Y., Bauer N., Knight L.M., Li Z., Liu S., Anderson C.J., Conti P.S., Sutcliffe J.L. (2014). Characterization and Evaluation of 64Cu-Labeled A20FMDV2 Conjugates for Imaging the Integrin αvβ6. Mol. Imaging Biol..

[39] Hausner S.H., Bauer N., Hu L.Y., Knight L.M., Sutcliffe J.L. (2015). The Effect of Bi-Terminal PEGylation of an Integrin αvβ6–Targeted ^18^F Peptide on Pharmacokinetics and Tumor Uptake. J. Nucl. Med..

[40] Satpati D., Bauer N., Hausner S.H., Sutcliffe J.L. (2014). Synthesis of [64Cu]DOTA-ADIBON3-Ala-PEG28-A20FMDV2 via Copper-Free Click Chemistry for PET Imaging of Integrin αvβ6. J. Radioanal. Nucl. Chem..

[41] Huynh T.T., Sreekumar S., Mpoy C., Rogers B.E. (2022). A Comparison of 64 Cu-Labeled Bi-Terminally PEGylated A20FMDV2 Peptides Targeting Integrin αvβ6. Oncotarget.

[42] Huynh T.T., Sreekumar S., Mpoy C., Rogers B.E. (2022). Therapeutic Efficacy of 177Lu-Labeled A20FMDV2 Peptides Targeting αvβ6. Pharmaceuticals.

[43] Rostovtsev V.V., Green L.G., Fokin V.V., Sharpless K.B. (2002). A Stepwise Huisgen Cycloaddition Process: Copper(I)-Catalyzed Regioselective “Ligation” of Azides and Terminal Alkynes. Angew. Chem. Int. Ed..

[44] Tornøe C.W., Christensen C., Meldal M. (2002). Peptidotriazoles on Solid Phase:  [1,2,3]-Triazoles by Regiospecific Copper(I)-Catalyzed 1,3-Dipolar Cycloadditions of Terminal Alkynes to Azides. J. Org. Chem..

[45] Breugst M., Reissig H.-U. (2020). The Huisgen Reaction: Milestones of the 1,3-Dipolar Cycloaddition. Angew. Chem. Int. Ed..

[46] Hausner S.H., Carpenter R.D., Bauer N., Sutcliffe J.L. (2013). Evaluation of an Integrin αvβ6-Specific Peptide Labeled with [18F]Fluorine by Copper-Free, Strain-Promoted Click Chemistry. Nucl. Med. Biol..

[47] Hausner S.H., Bold R.J., Cheuy L.Y., Chew H.K., Daly M.E., Davis R.A., Foster C.C., Kim E.J., Sutcliffe J.L. (2019). Preclinical Development and First-in-Human Imaging of the Integrin αvβ6 with [^18^F]αvβ6-Binding Peptide in Metastatic Carcinoma. Clin. Cancer Res..

[48] Mitchell A.R., Kent S.B.H., Engelhard M., Merrifield R.B. (1978). A New Synthetic Route to Tert-Butyloxycarbonylaminoacyl-4-(Oxymethyl)Phenylacetamidomethyl-Resin, an Improved Support for Solid-Phase Peptide Synthesis. J. Org. Chem..

[49] Harris P.W.R., Yang S.H., Brimble M.A. (2011). An Improved Procedure for the Preparation of Aminomethyl Polystyrene Resin and Its Use in Solid Phase (Peptide) Synthesis. Tetrahedron Lett..

[50] Hausner S.H., DiCara D., Marik J., Marshall J.F., Sutcliffe J.L. (2007). Use of a Peptide Derived from Foot-and-Mouth Disease Virus for the Noninvasive Imaging of Human Cancer: Generation and Evaluation of 4-[^18^F]Fluorobenzoyl A20FMDV2 for in vivo Imaging of Integrin αvβ6 Expression with Positron Emission Tomography. Cancer Res..

[51] John A.E., Luckett J.C., Tatler A.L., Awais R.O., Desai A., Habgood A., Ludbrook S., Blanchard A.D., Perkins A.C., Jenkins R.G. (2013). Preclinical SPECT/CT Imaging of αvβ6 Integrins for Molecular Stratification of Idiopathic Pulmonary Fibrosis. J. Nucl. Med..

[52] Arano Y., Uezono T., Akizawa H., Ono M., Wakisaka K., Nakayama M., Sakahara H., Konishi J., Yokoyama A. (1996). Reassessment of Diethylenetriaminepentaacetic Acid (DTPA) as a Chelating Agent for Indium-111 Labeling of Polypeptides Using a Newly Synthesized Monoreactive DTPA Derivative. J. Med. Chem..

[53] Walter R., Neidle A., Marks N. (1975). Significant Differences in the Degradation of Pro-Leu-Gly-NH_2_ by Human Serum and That of Other Species. Proc. Soc. Exp. Biol. Med..

[54] Benuck M., Marks N. (1976). Differences in the Degradation of Hypothalamic Releasing Factors by Rat and Human Serum. Life Sci..

[55] Witter A., Scholtens H., Verhoef J. (1980). H-Pro-[3H]Leu-Gly-NH2: Metabolism in Human and Rat Plasma Investigated by High-Pressure Liquid Chromatography. Neuroendocrinology.

[56] McDermott J.R., Smith A.I., Biggins J.A., Hardy J.A., Dodd P.R., Edwardson J.A. (1981). Degradation of Luteinizing Hormone-Releasing Hormone by Serum and Plasma in vitro. Regul. Pept..

[57] Powell M.F., Grey H., Gaeta F., Sette A., Colón S. (1992). Peptide Stability in Drug Development: A Comparison of Peptide Reactivity in Different Biological Media. J. Pharm. Sci..

[58] Jenssen H., Aspmo S.I., Otvos L. (2008). Serum Stability of Peptides. Peptide-Based Drug Design.

[59] Kogelberg H., Tolner B., Thomas G.J., Di Cara D., Minogue S., Ramesh B., Sodha S., Marsh D., Lowdell M.W., Meyer T. (2008). Engineering a Single-Chain Fv Antibody to αvβ6 Integrin Using the Specificity-Determining Loop of a Foot-and-Mouth Disease Virus. J. Mol. Biol..

