# Supplementary material for: Synthesis and Systematic Study on the Effect of Different PEG Units on Stability of PEGylated, Integrin-αvβ6-Specific A20FMDV2 Analogues in Rat Serum and Human Plasma
Source: Molecules. 2022 Jul 6;27(14):4331. doi: 10.3390/molecules27144331 (PMC9316855; doi:10.3390/molecules27144331)
Supplement: Supplementary file 1 [file molecules-27-04331-s001.zip › molecules-172276-supplementary.pdf]

molecules

Supplementary Information

Synthesis and Systematic Study on the Effect of Different PEG units on Stability of PEGylated, Integrin αvβ6-Specific A20FMDV2 Analogues in Rat Serum and Human Plasma

Kuo-yuan Hung [1], Renata Kowalczyk [1,*], Ami Desai [2], Margaret A. Brimble [1,3,4], John F. Marshall [2,*] and Paul W. R. Harris [1,3,4,*]

1 The School of Chemical Sciences, University of Auckland, 23 Symonds St, Auckland 1010, New Zealand; hungkuoyuan@gmail.com (K.-y.H.); r.kowalczyk@auckland.ac.nz (R.K.); m.brimble@auckland.ac.nz (M.A.B.)
2 Centre for Tumour Biology, Barts Cancer Institute-Cancer Research UK Centre of Excellence, Queen Mary University of London, Charterhouse Square, London, UK; ami_desai89@hotmail.com (A.D.)
3 Maurice Wilkins Centre for Molecular Biodiscovery, University of Auckland, Private Bag 92019, Auckland 1010, New Zealand
4 The School of Biological Sciences, University of Auckland, 3A Symonds St, Auckland 1010, New Zealand
* Correspondence: j.f.marshall@qmul.ac.uk (J.F.M.); paul.harris@auckland.ac.nz (P.W.R.H.)

Structure, RP-HPLC and MS profile of synthetic peptides 13-30

NAVPNLRGDLQVLAQKVART-OH (13) (A20FMDV2)

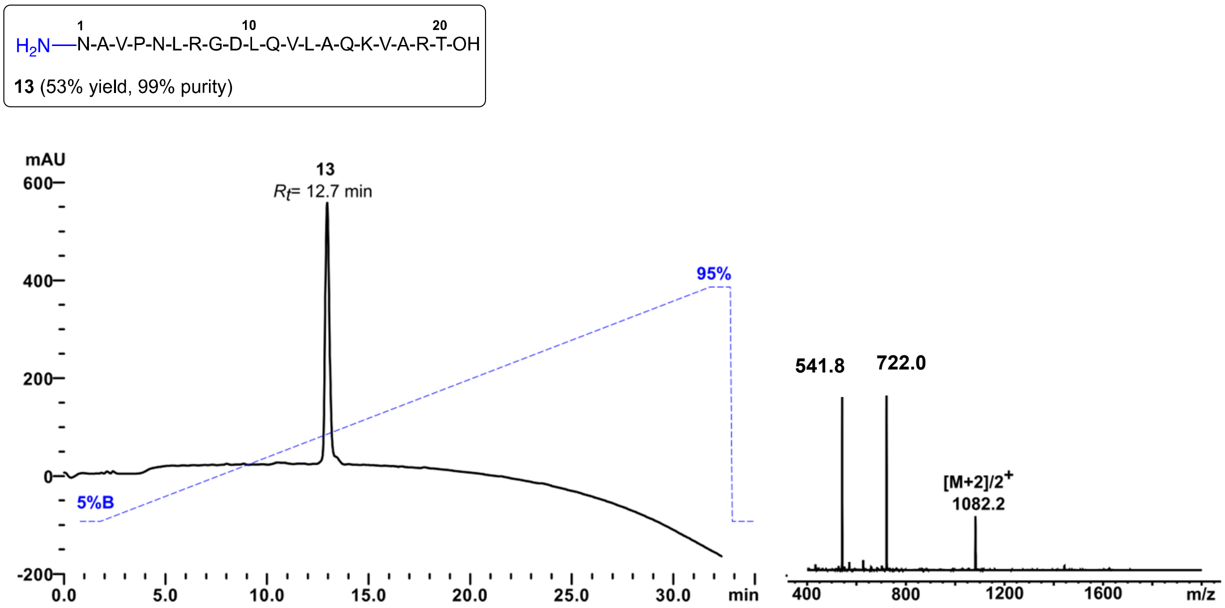

Figure S1. HPLC and MS traces of pure peptide 13 (*ca* 99% as judged by peak area of RP-HPLC at 210 nm, 53% yield). R_t 12.7 min; *m/z* 541.8 $[M + 4H]^{4+}$ requires 541.9, *m/z* 722.0 $[M + 3H]^{3+}$ requires 722.2, *m/z* 1082.2 $[M + 2H]^{2+}$ requires 1082.8. Mass deconvolution calculated at 2162.87 Da with standard deviation of 0.42; theoretical mass calculated at 2163.50 Da.

H_2N-PEG$_1$-CH_2CO-NAVPNLRGDLQVLAQKVART-OH (14)

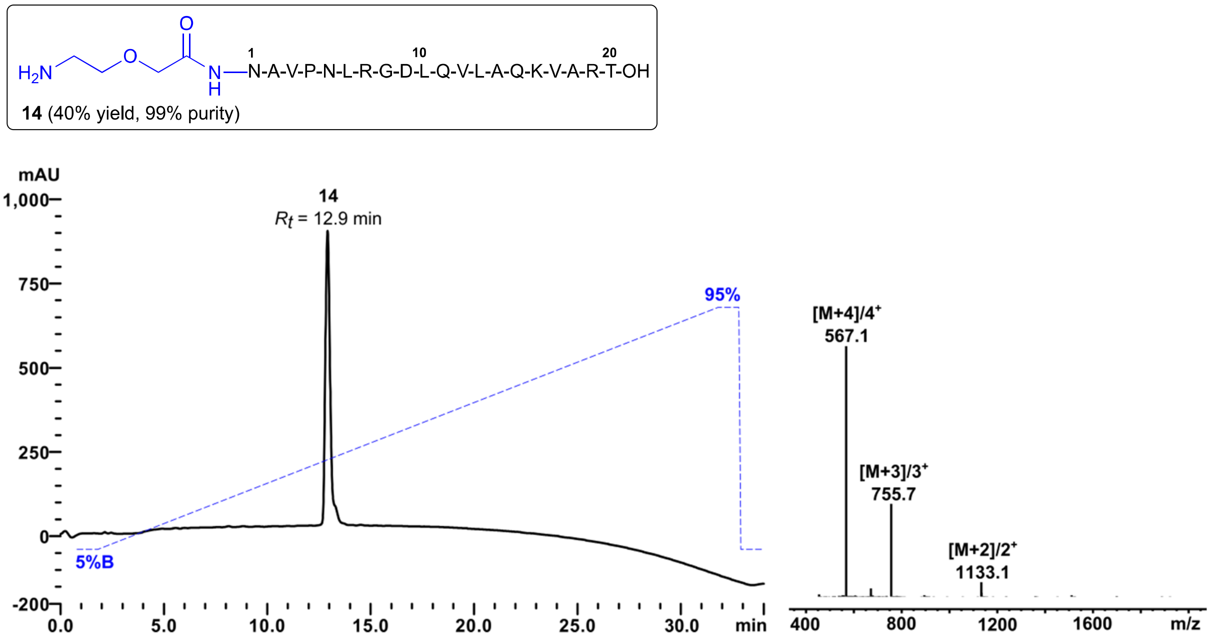

Figure S2. HPLC and MS traces of pure peptide 14 (*ca* 99% as judged by peak area of RP-HPLC at 210 nm, 40% yield). R_t 12.9 min; *m/z* 567.1 $[M + 4H]^{4+}$ requires 567.2, *m/z* 755.7 $[M + 3H]^{3+}$ requires 755.9, *m/z* 1133.1 $[M + 2H]^{2+}$ requires 1133.3. Mass deconvolution calculated at 2264.23 Da with standard deviation of 0.15; theoretical mass calculated at 2264.62 Da.

H_2N-PEG$_2$-CH_2CO-NAVPNLRGDLQVLAQKVART-OH (15)

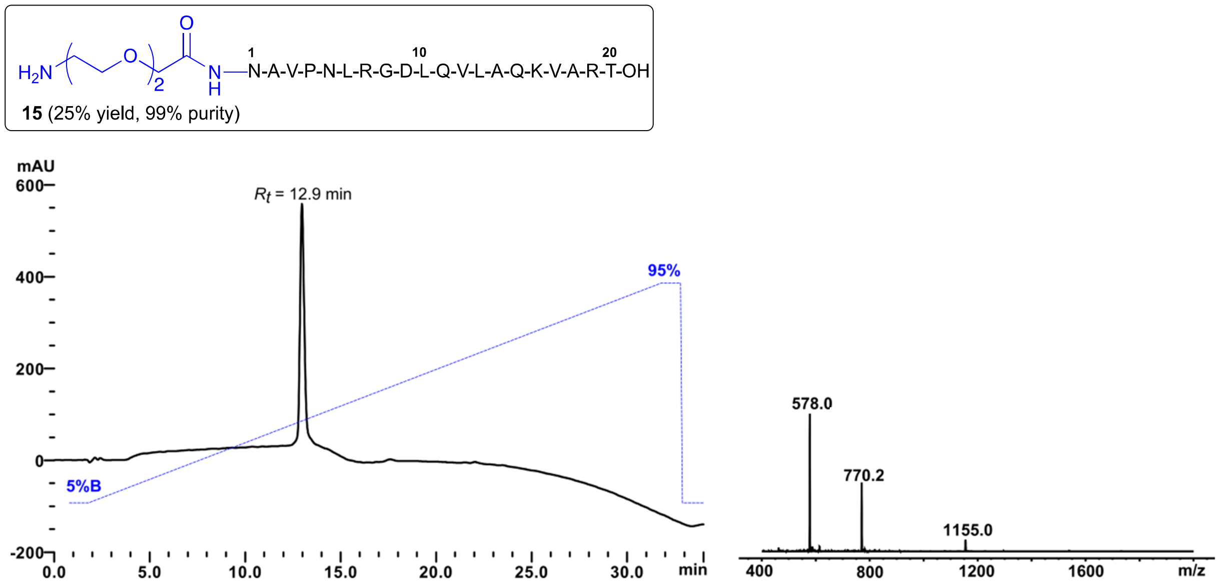

Figure S3. HPLC and MS traces of pure peptide 15 (*ca* 99% as judged by peak area of RP-HPLC at 210 nm, 25% yield). R_t 12.9 min; *m*/*z* 578.0 $[M + 4H]^{4+}$ requires 578.2, *m*/*z* 770.2 $[M + 3H]^{3+}$ requires 770.6, *m*/*z* 1155.0 $[M + 2H]^{2+}$ requires 1155.3. Mass deconvolution calculated at 2307.87 Da with standard deviation of 0.23; theoretical mass calculated at 2308.67 Da.

H_2N-PEG$_3$-CH_2CO-NAVPNLRGDLQVLAQKVART-OH (16)

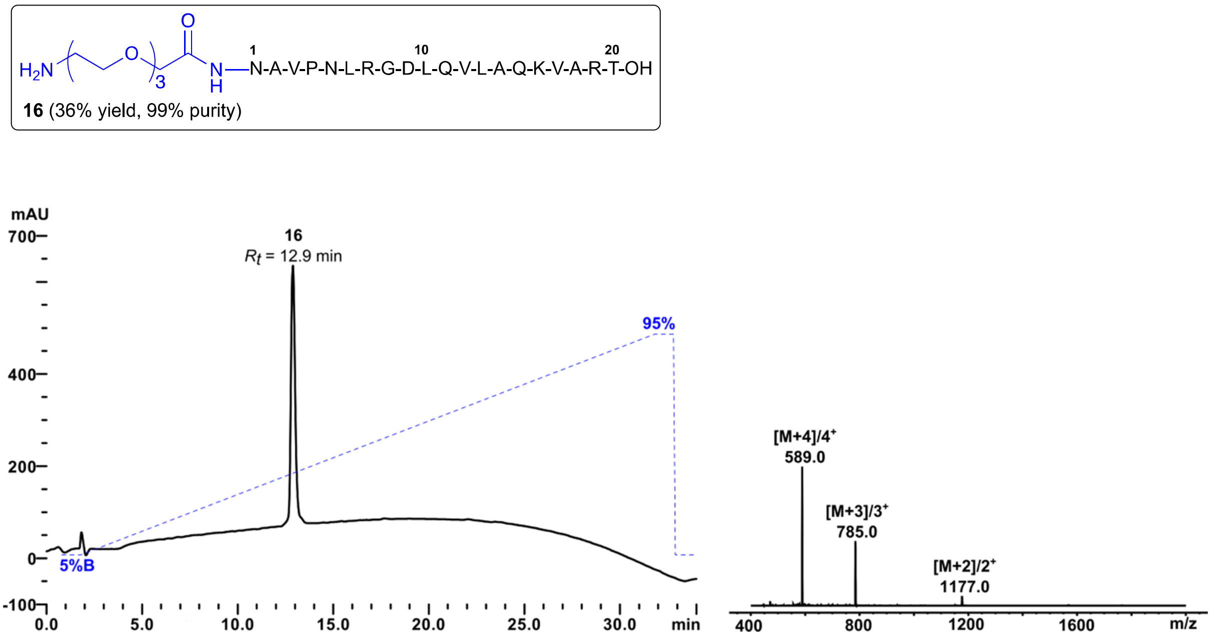

Figure S4. HPLC and MS traces of pure peptide 16 (*ca* 99% as judged by peak area of RP-HPLC at 210 nm, 36% yield). R_t 12.9 min; *m*/*z* 589.0 $[M + 4H]^{4+}$ requires 589.2, *m*/*z* 785 $[M + 3H]^{3+}$ requires 785.3, *m*/*z* 1177.0 $[M + 2H]^{2+}$ requires 1177.4. Mass deconvolution calculated at 2352.00 Da with standard deviation of 0.00; theoretical mass calculated at 2352.73 Da.

H_2N-PEG$_4$-CH_2CO-NAVPNLRGDLQVLAQKVART-OH (17)

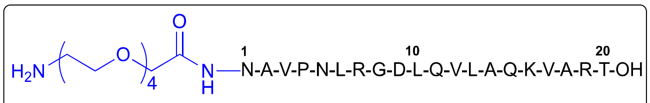

17 (40% yield, 99% purity)

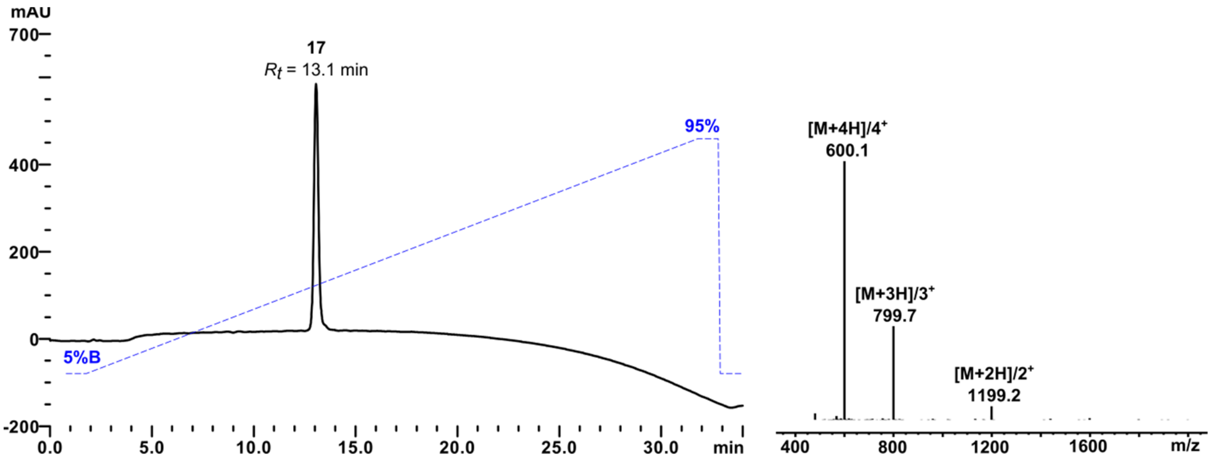

Figure S5. HPLC and MS traces of pure peptide 17 (*ca* 99% as judged by peak area of RP-HPLC at 210 nm, 40% yield). R_t 13.1 min; *m/z* 600.1 $[M + 4H]^{4+}$ requires 600.2, *m/z* 799.7 $[M + 3H]^{3+}$ requires 799.9, *m/z* 1199.2 $[M + 2H]^{2+}$ requires 1199.4. Mass deconvolution calculated at 2396.30 Da with standard deviation of 0.17; theoretical mass calculated at 2396.78 Da.

H_2N-PEG$_5$-CH_2CO-NAVPNLRGDLQVLAQKVART-OH (18)

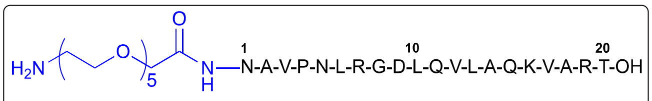

18 (38% yield, 99% purity)

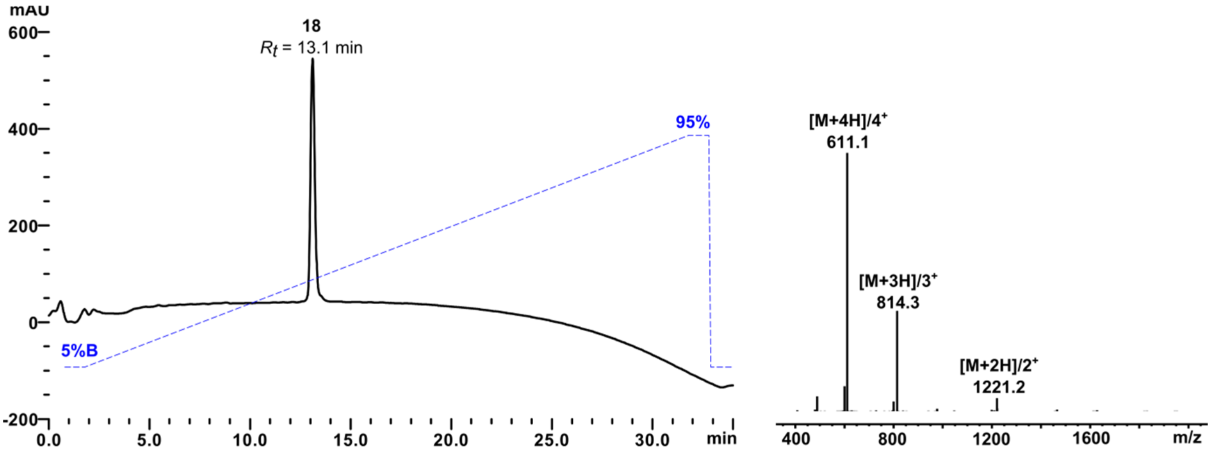

Figure S6. HPLC and MS traces of pure peptide 18 (*ca* 99% as judged by peak area of RP-HPLC at 210 nm, 38% yield). R_t 13.1 min; *m/z* 611.1 $[M + 4H]^{4+}$ requires 611.2, *m/z* 814.3 $[M + 3H]^{3+}$ requires 814.6, *m/z* 1221.2 $[M + 2H]^{2+}$ requires 1221.4. Mass deconvolution calculated at 2440.23 Da with standard deviation of 0.29; theoretical mass calculated at 2440.83 Da.

H_2N-PEG$_8$-CH_2CO-NAVPNLRGDLQVLAQKVART-OH (19)

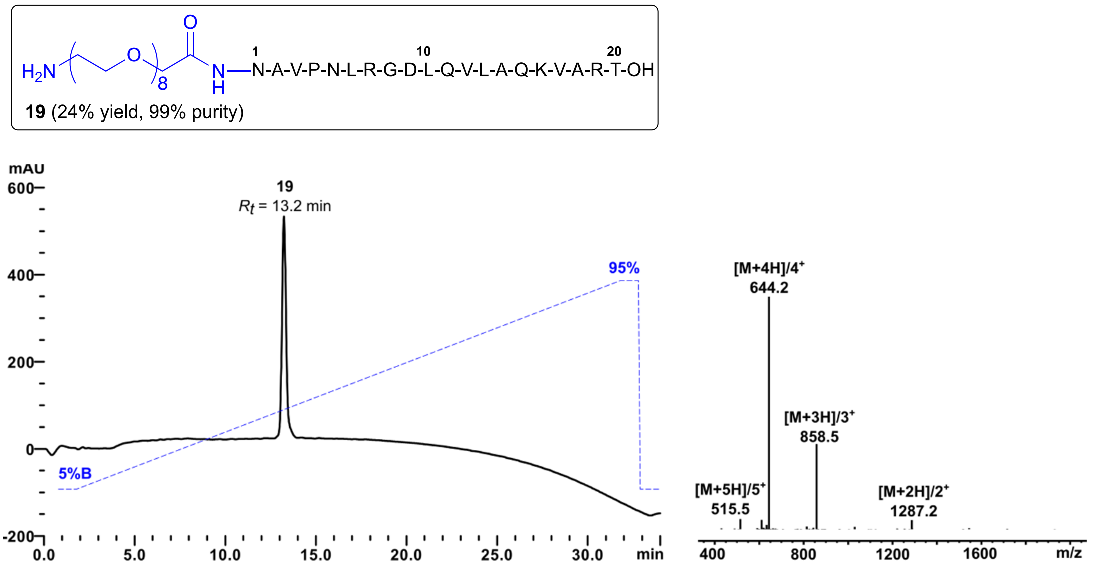

Figure S7. HPLC and MS traces of pure peptide 19 (*ca* 99% as judged by peak area of RP-HPLC at 210 nm, 24% yield). R_t 13.2 min; *m/z* 515.5 $[M + 5H]^{5+}$ requires 515.6, *m/z* 644.2 $[M + 4H]^{4+}$ requires 644.3, *m/z* 858.5 $[M + 3H]^{3+}$ requires 858.7, *m/z* 1287.2 $[M + 2H]^{2+}$ requires 1287.5. Mass deconvolution calculated at 2572.55 Da with standard deviation of 0.17; theoretical mass calculated at 2572.99 Da.

H_2N-PEG$_5$-CH_2CH_2CO-NAVPNLRGDLQVLAQKVART-OH (20)

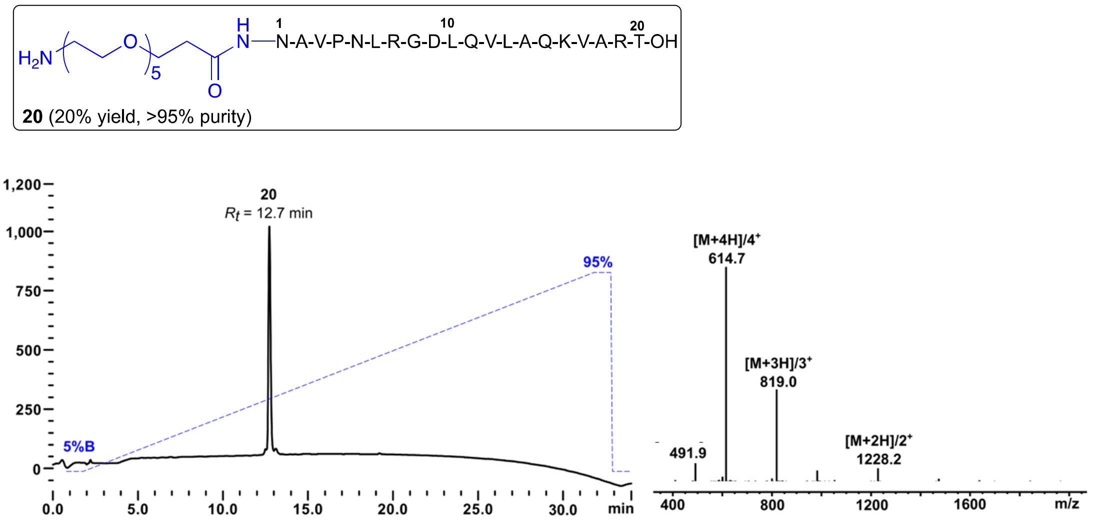

Figure S8. HPLC and MS traces of pure peptide 20 (>95% as judged by peak area of RP-HPLC at 210 nm, 20% yield). R_t 12.7 min; *m/z* 491.9 $[M + 5H]^{5+}$ requires 492.0, *m/z* 614.7 $[M + 4H]^{4+}$ requires 614.7, *m/z* 819.0 $[M + 3H]^{3+}$ requires 819.3, *m/z* 1228.2 $[M + 2H]^{2+}$ requires 1228.4. Mass deconvolution calculated at 2454.43 Da with standard deviation of 0.17; theoretical mass calculated at 2454.86 Da.

H_2N-PEG_{10}-CH_2CH_2CO-NAVPNLRGDLQVLAQKVART-OH (21)

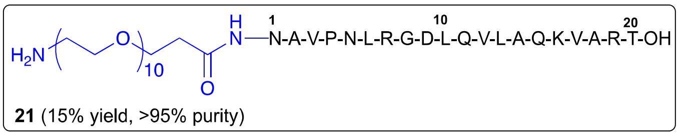

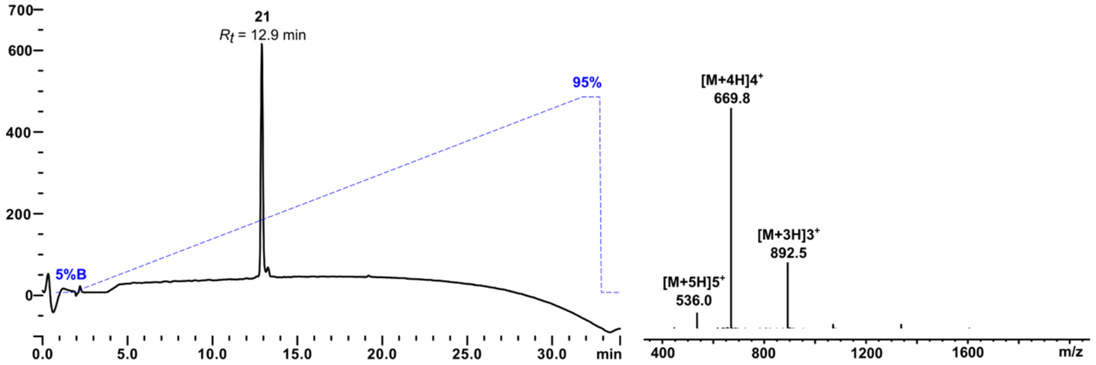

Figure S9. HPLC and MS traces of pure peptide 21 (>95% as judged by peak area of RP-HPLC at 210 nm, 15% yield). R_t 12.9 min; *m*/*z* 536.0 $[M + 5H]^{5+}$ requires 536.0, *m*/*z* 669.8 $[M + 4H]^{4+}$ requires 669.8, *m*/*z* 892.5 $[M + 3H]^{3+}$ requires 892.7. Mass deconvolution calculated at 2674.90 Da with standard deviation of 0.36; theoretical mass calculated at 2675.12 Da.

H_2N-PEG_{15}-CH_2CH_2CO-NAVPNLRGDLQVLAQKVART-OH (22)

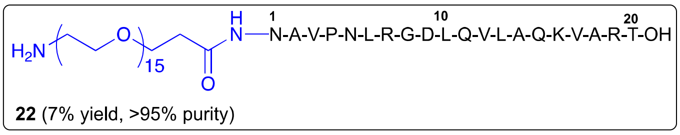

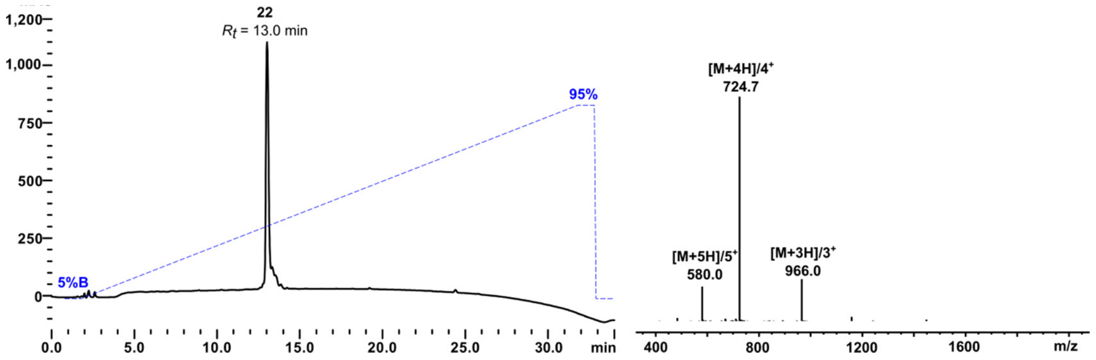

Figure S10. HPLC and MS traces of pure peptide 22 (>95% as judged by peak area of RP-HPLC at 210 nm, 7% yield). R_t 13.0 min; *m*/*z* 580.0 $[M + 5H]^{5+}$ requires 580.1, *m*/*z* 724.7 $[M + 4H]^{4+}$ requires 724.8, *m*/*z* 966.0 $[M + 3H]^{3+}$ requires 966.1. Mass deconvolution calculated at 2894.93 Da with standard deviation of 0.12; theoretical mass calculated at 2895.36 Da.

H_2N-PEG$_{20}$-CH_2CH_2CO-NAVPNLRGDLQVLAQKVART-OH (23)

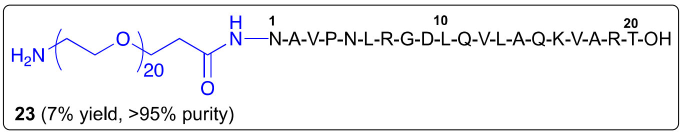

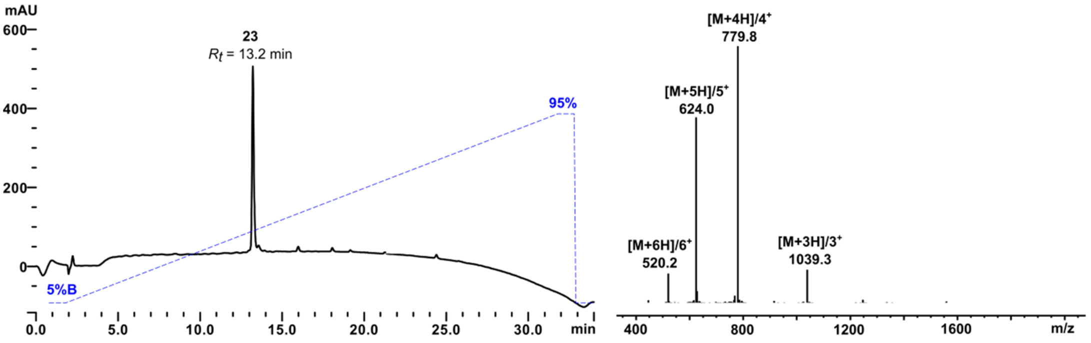

Figure S11. HPLC and MS traces of pure peptide 23 (>95% as judged by peak area of RP-HPLC at 210 nm, 7% yield). R_t 13.2 min; *m*/*z* 520.2 [M + 6H]$^{6+}$ requires 520.3, *m*/*z* 624.0 [M + 5H]$^{5+}$ requires 624.1, *m*/*z* 779.8 [M + 4H]$^{4+}$ requires 779.9, *m*/*z* 1039.3 [M + 3H]$^{3+}$ requires 1039.6. Mass deconvolution calculated at 3115.08 Da with standard deviation of 0.15; theoretical mass calculated at 3115.65 Da.

DTPA-NK(D-biotin)VPNLRGDLQVLAQKVART-OH (24)

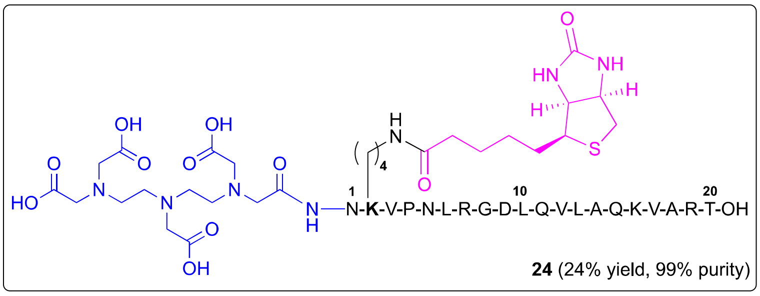

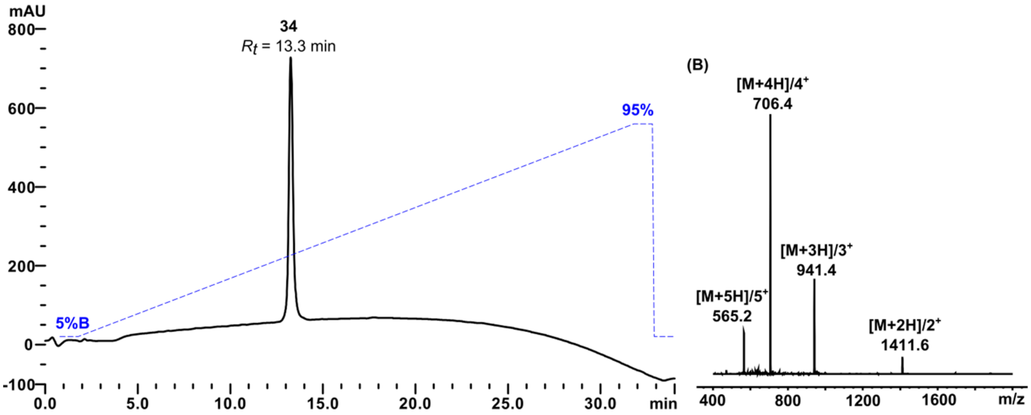

Figure S12. HPLC and MS traces of pure peptide 24 (*ca* 99% as judged by peak area of RP-HPLC at 210 nm, 24% yield). R_t 13.3 min; *m*/*z* 565.2 [M + 5H]$^{5+}$ requires 565.2, *m*/*z* 706.4 [M + 4H]$^{4+}$ requires 706.2, *m*/*z* 941.4 [M + 3H]$^{3+}$ requires 941.3, *m*/*z* 1411.6 [M + 2H]$^{2+}$ requires 1411.4. Mass deconvolution calculated at 2821.25 Da with standard deviation of 0.25; theoretical mass calculated at 2820.77 Da.

DTPA-PEG2-CH2CO-NK(D-biotin)VPNLRGDLQVLAQKVART-OH (25)

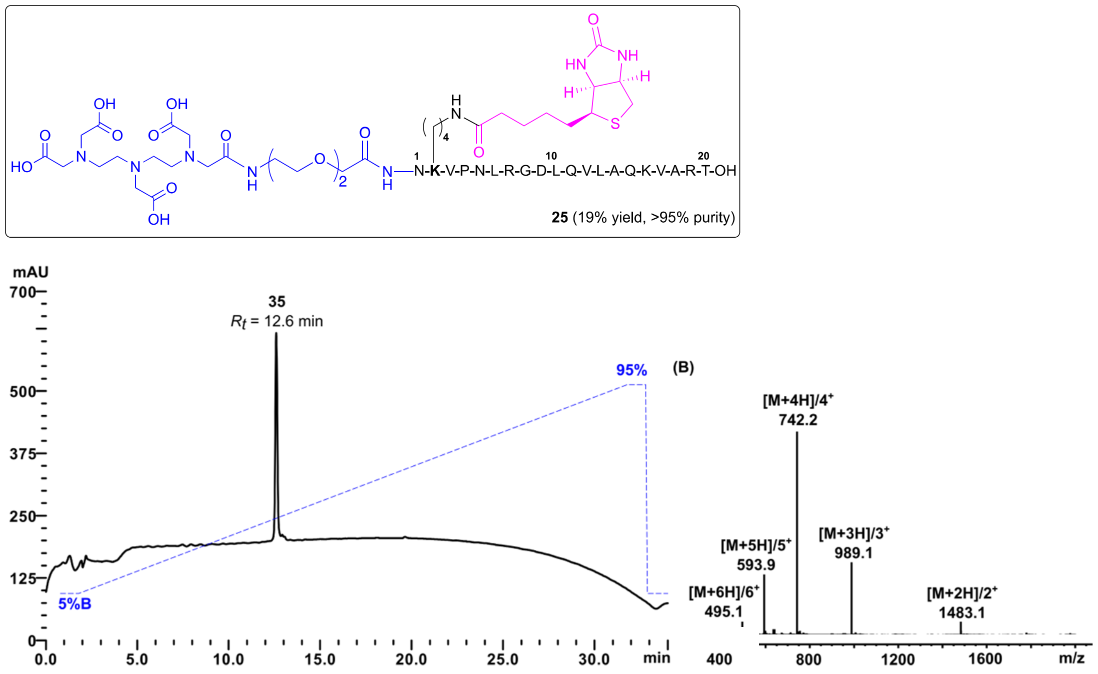

Figure S13. HPLC and MS traces of pure peptide 25 (>95% as judged by peak area of RP-HPLC at 210 nm, 19% yield). R_t 12.6 min; *m/z* 495.1 $[M + 6H]^{6+}$ requires 495.6, *m/z* 593.9 $[M + 5H]^{5+}$ requires 594.5, *m/z* 742.2 $[M + 4H]^{4+}$ requires 742.9, *m/z* 989.1 $[M + 3H]^{3+}$ requires 990.1, *m/z* 1483.1 $[M + 2H]^{2+}$ requires 1484.7. Mass deconvolution calculated at 2964.48 Da with standard deviation of 0.24; theoretical mass calculated at 2967.42 Da.

DTPA-PEG5-CH2CO-NK(D-biotin)VPNLRGDLQVLAQKVART-OH (26)

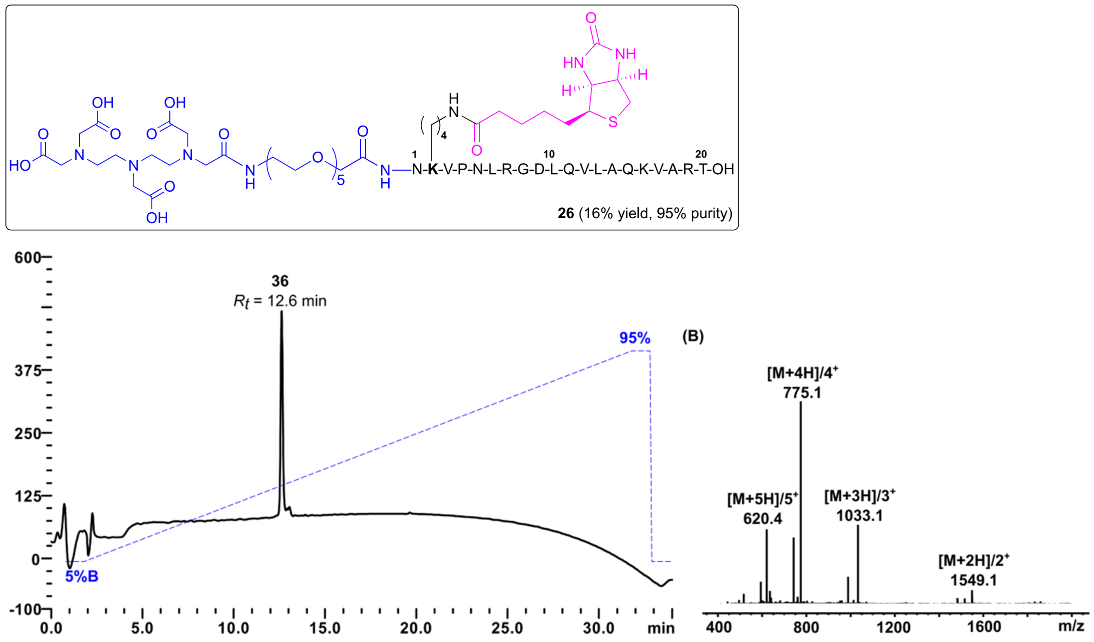

Figure S14. HPLC and MS traces of pure peptide 26 (*ca* 95% as judged by peak area of RP-HPLC at 210 nm, 16% yield). R_t 12.6 min; *m/z* 620.4 $[M + 5H]^{5+}$ requires 620.7, *m/z* 775.1 $[M + 4H]^{4+}$ requires 775.7, *m/z* 1033.1 $[M + 3H]^{3+}$ requires 1033.9, *m/z* 1549.1 $[M + 2H]^{2+}$ requires 1550.3. Mass deconvolution calculated at 3096.48 Da with standard deviation of 0.36; theoretical mass calculated at 3098.58 Da.

DTPA-PEG$_5$-CH$_2$CH$_2$CO-NK(D-biotin)VPNLRGDLQVLAQKVART-OH (27)

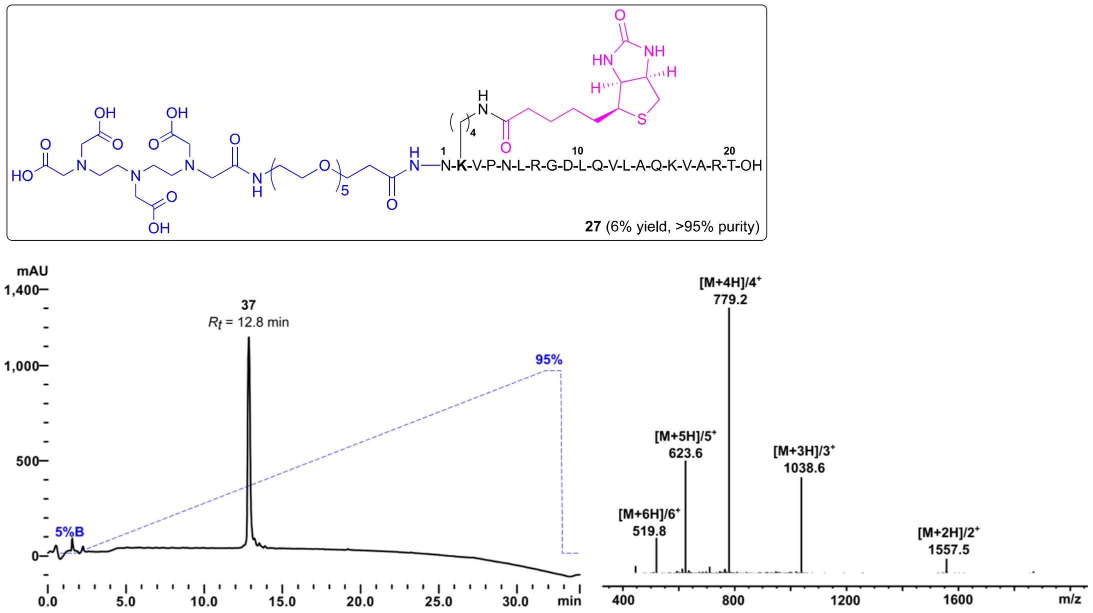

Figure S15. HPLC and MS traces of pure peptide 27 (>95% as judged by peak area of RP-HPLC at 210 nm, 6% yield). R_t 12.8 min; *m/z* 519.8 [M + 6H]$^{6+}$ requires 519.9, *m/z* 623.6 [M + 5H]$^{5+}$ requires 623.7, *m/z* 779.2 [M + 4H]$^{4+}$ requires 779.4, *m/z* 1038.6 [M + 3H]$^{3+}$ requires 1038.9, *m/z* 1557.5 [M + 2H]$^{2+}$ requires 1557.8. Mass deconvolution calculated at 3112.88 Da with standard deviation of 0.11; theoretical mass calculated at 3113.61 Da.

DTPA-PEG$_{10}$-CH$_2$CH$_2$CO-NK(D-biotin)VPNLRGDLQVLAQKVART-OH (28)

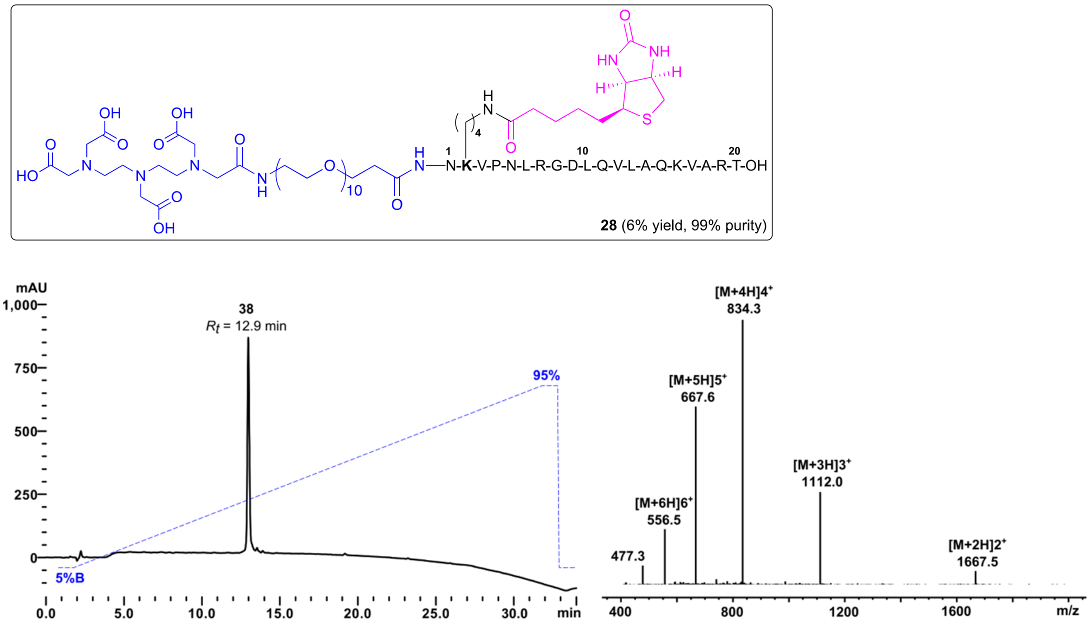

Figure S16. HPLC and MS traces of pure peptide 28 (*ca* 99% as judged by peak area of RP-HPLC at 210 nm, 6% yield). R_t 12.9 min; *m/z* 477.3 [M + 7H]$^{7+}$ requires 477.3, *m/z* 556.5 [M + 6H]$^{6+}$ requires 556.6, *m/z* 667.6 [M + 5H]$^{5+}$ requires 667.8, *m/z* 834.3 [M + 4H]$^{4+}$ requires 834.5, *m/z* 1112.0 [M + 3H]$^{3+}$ requires 1112.3, *m/z* 1667.5 [M + 2H]$^{2+}$ requires 1667.9. Mass deconvolution calculated at 3333.04 Da with standard deviation of 0.11; theoretical mass calculated at 3333.87 Da.

DTPA-PEG$_{15}$-CH$_2$CH$_2$CO-NK(D-biotin)VPNLRGDLQVLAQKVART-OH (29)

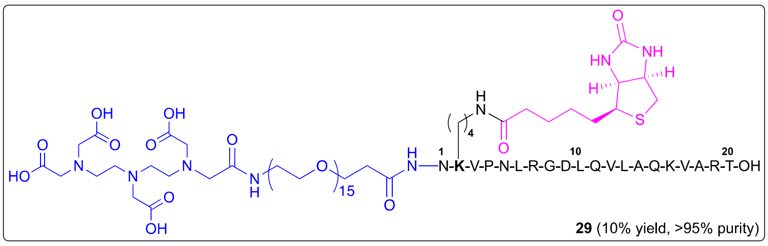

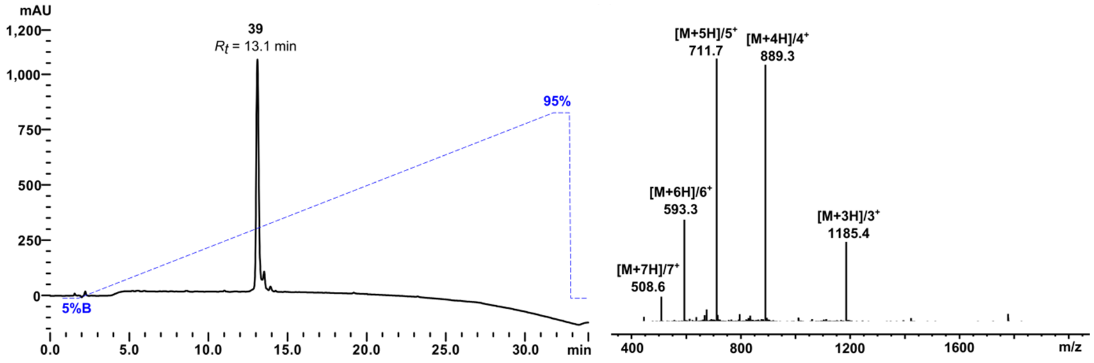

Figure S17. HPLC and MS traces of pure peptide 29 (>95% as judged by peak area of RP-HPLC at 210 nm, 10% yield). R_t 13.1 min; *m/z* 508.6 [M + 7H]$^{7+}$ requires 508.7, *m/z* 593.3 [M + 6H]$^{6+}$ requires 593.4, *m/z* 711.7 [M + 5H]$^{5+}$ requires 711.8, *m/z* 889.3 [M + 4H]$^{4+}$ requires 889.5, *m/z* 1185.4 [M + 3H]$^{3+}$ requires 1185.7. Mass deconvolution calculated at 3553.43 Da with standard deviation of 0.29; theoretical mass calculated at 3554.11 Da.

DTPA-PEG$_{20}$-CH$_2$CH$_2$CO-NK(D-biotin)VPNLRGDLQVLAQKVART-OH (30)

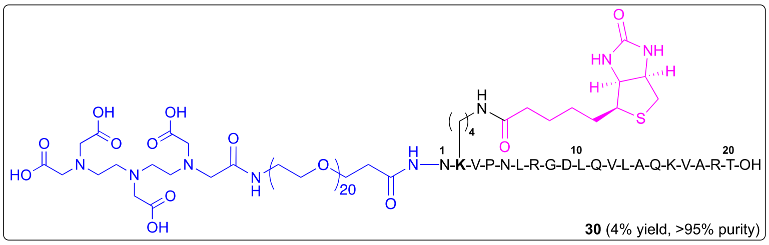

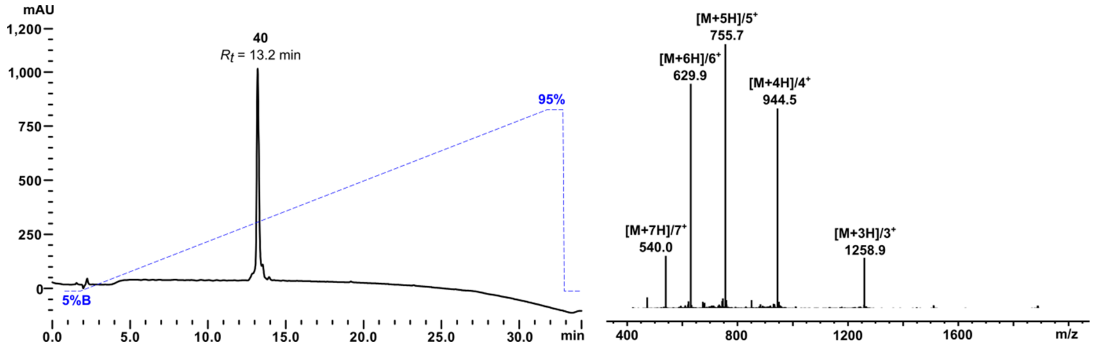

Figure S18. HPLC and MS traces of pure peptide 30 (>95% as judged by peak area of RP-HPLC at 210 nm, 4% yield). R_t 13.2 min; *m/z* 540.0 [M + 7H]$^{7+}$ requires 540.2, *m/z* 629.9 [M + 6H]$^{6+}$ requires 630.1, *m/z* 755.7 [M + 5H]$^{5+}$ requires 755.9, *m/z* 944.5 [M + 4H]$^{4+}$ requires 944.6, *m/z* 1258.9 [M + 3H]$^{3+}$ requires 1259.1. Mass deconvolution calculated at 3773.65 Da with standard deviation of 0.26; theoretical mass calculated at 3774.40 Da.
